# Photoaging enhances the leaching of di(2-ethylhexyl) phthalate and transformation products from polyvinyl chloride microplastics into aquatic environments

**DOI:** 10.1038/s42004-024-01310-3

**Published:** 2024-09-27

**Authors:** Charlotte Henkel, Thorsten Hüffer, Ruoting Peng, Xiaoyu Gao, Subhasis Ghoshal, Thilo Hofmann

**Affiliations:** 1 University of Vienna, Centre for Microbiology and Environmental Systems Science, Department for Environmental Geosciences, Josef-Holaubek-Platz 2, 1090 Vienna, Austria; 2 University of Vienna, Doctoral School in Microbiology and Environmental Science, Djerassiplatz 1, 1030 Vienna, Austria; 3 University of Vienna, Research Platform Plastics in the Environment and Society (Plenty), Josef-Holaubek-Platz 2, 1090 Vienna, Austria; 4 McGill University, Department of Civil Engineering, 817 Sherbrooke Street West, Montreal, Quebec H3A 0C3 Canada

**Keywords:** Environmental chemistry, Organic chemistry, Pollution remediation, Polymer characterization

## Abstract

Increasing chemical pollution is a threat to sustainable water resources worldwide. Plastics and harmful additives released from plastics add to this burden and might pose a risk to aquatic organisms, and human health. Phthalates, which are common plasticizers and endocrine-disrupting chemicals, are released from polyvinyl chloride (PVC) microplastics and are a cause of concern. Therefore, the leaching kinetics of additives, including the influence of environmental weathering, are key to assessing exposure concentrations but remain largely unknown. We show that photoaging strongly enhances the leaching rates of di(2-ethylhexyl) phthalate (DEHP) by a factor of 1.5, and newly-formed harmful transformation products, such as mono(2-ethylhexyl) phthalate (MEHP), phthalic acid, and phthalic anhydride from PVC microplastics into the aquatic environment. Leaching half-lives of DEHP reduced from 449 years for pristine PVC to 121 years for photoaged PVC. Aqueous boundary layer diffusion (ABLD) is the limiting mass transfer process for the release of DEHP from pristine and photoaged PVC microplastics. The leaching of transformation products is limited by intraparticle diffusion (IPD). The calculated mass transfer rates can be used to predict exposure concentrations of harmful additives in the aquatic environment.

## Abstract (web summary)

The environmental weathering of plastics and the leaching kinetics of additives are key to assessing exposure concentrations. Here, the authors show that photoaging enhances the leaching rate of the common additive di(2-ethylhexyl) phthalate (DEHP) by a factor of 1.5, and newly-formed harmful transformation products, such as mono(2-ethylhexyl) phthalate (MEHP), phthalic acid, and phthalic anhydride, are released from PVC microplastics into the aquatic environment.

## Introduction

Global chemical pollution is an issue of increasing concern^1^. Rapidly increasing numbers and amounts of chemicals are being released into the environment through various sources with possibly detrimental consequences for sustainable living in the future^2,3^. Plastics and potentially toxic substances stemming from plastics add to this chemical burden and are a threat to humans and animal life^4,5^. In the environment, plastics are subject to degradation and fragmentation processes, leading to the formation of ever smaller particles of various shapes, structures, and sizes, including microplastics (<5000 µm)^6^. Microplastics are ubiquitous in the environment and have become an increasing, multifaceted problem for living organisms and human health. On a global scale, microplastics can negatively impact carbon and nutrient cycling (including nitrogen and phosphorous) in aquatic environments, and they can irreversibly alter the structure of sediments and soil habitats^7^. At the individual level, exposure to microplastics through uptake (e.g., ingestion and inhalation) or skin contact can cause serious health damage, including inflammation and oxidative stress in humans and animals^8,9^.

In addition to physical hazards, (micro)plastics contain a plethora of chemical compounds. These include non-intentionally added substances (NIAS) such as by-products, degradation products, and contaminants^10^. In addition to NIAS, which are introduced at all stages of the life cycle of plastics, more than 10,000 chemicals such as monomers, processing aids, and additives (55% of the identified chemicals) are used intentionally for the manufacturing of plastic products. More than 2400 of these intentionally used plastic chemicals are potentially hazardous given their persistent, bio-accumulative, toxic, or endocrine-disruptive properties^11^. Additives contribute to improving the material properties of plastics and can account for up to 70 wt% of plastic products for PVC^5,12^. With few exceptions, additives are not chemically bound to the polymer and therefore inevitably leach into the surrounding environment during the life-span of the plastic product^5,13^. Some additives are even designed to migrate through plastics and be released^14^.

Potentially harmful additives used in plastic manufacturing can become a serious problem in the environment and for humans^15^. For example, the release of a tire rubber-derived additive, i.e., its transformation product (6PPDq), caused acute recurrent mortality of coho salmon populations in urban creeks^14^. Bisphenol A (BPA) and its analogs bisphenol S and bisphenol F interfere with the hormone system^16^. In particular, exposure to plasticizers, more specifically, phthalic acid esters (phthalates), has been associated with several health damages arising from their endocrine-disrupting effects and linked diseases such as liver cancer, type II diabetes, and reproductive injuries^15^. Phthalates, e.g., benzyl butyl phthalate (BBP), dibutyl phthalate (DPB), and di(2-ethylhexyl) phthalate (DEHP), are known to have adverse health effects^17^, but they have nonetheless been found in various everyday plastic products, including food contact materials^18^ and medical supplies^19^. Worldwide, 6–8 million tons of phthalates are produced annually, with the main use (80%) being in the manufacture of polyvinyl chloride (PVC)^5,20^. Recently, the European Chemicals Agency (ECHA) has specifically identified the release of ortho-phthalates such as DEHP from PVC as a risk to humans and the environment, with PVC microplastics being the main source of phthalate release^21^.

There is an urgent need to assess the chemical pollution stemming from additives, especially phthalates released from PVC microplastics. To assess the exposure and, ultimately, the risk for aquatic and human life, in-depth knowledge of leaching kinetics and processes is required to predict environmental concentrations of phthalates. Diffusion models, such as the intraparticle diffusion (IPD) model and the aqueous boundary layer diffusion (ABLD) model, have been applied to elucidate the leaching of organic contaminants and additives, for example, organochlorine pesticides^22^, polycyclic compounds^23^, polychlorinated biphenyls^24^, brominated flame retardants^25^, chlorinated benzenes^26^, and phthalates^27^ from different micron-sized polymers including polyethylene (PE), polypropylene (PP), and PVC into aqueous systems. Process-specific parameters for IPD and ABLD depend on the properties of the diffusing compound and the polymer^28^. The relative contribution of IPD and ABLD to the overall leaching process is specific to the contaminant-polymer combinations under consideration. In general, ABLD is the limiting mass transfer process for compounds with high octanol-water partition coefficients (K_O/W_) in combination with a high ABL thickness, and a short diffusion distance in the polymer^22^. This is exemplified by thin PE sheets, which are commonly used as passive samplers for hydrophobic organic contaminants in aqueous environments^29^. Under environmentally relevant conditions, the leaching of DEHP from pristine PVC into air^30^ and water^27^ is limited by BLD. In aquatic environments, however, several factors, including flow conditions, salinity, the aqueous concentration of organic carbon, and the water temperature, crucially influence leaching. The impact of these factors on the leaching kinetics and processes has been determined by integrating experimental data into models in our previous study^31^. In addition, weathering of plastics, for example, due to heat, light, mechanical stress, and microbial activity, requires consideration when investigating leaching processes^32^.

PVC is one of the least stable polymers in the environment^33,34^. During the manufacturing process, an often complex mixture of stabilizers is therefore added to the polymer to ensure its durability in outdoor applications^35^. In particular, PVC is susceptible to photodegradation by UV light with a peak sensitivity at 320 nm^36^. UV irradiation induces the autocatalytic dechlorination of PVC (Fig. S5)^34^. In the absence of oxygen, photochemical reactions of the polymer lead to the formation of polyenic -(CH=CH-)_n_- sequences and thus to the discoloration (yellowing) of the polymer. Under atmospheric conditions, the oxidation of polyene (photobleaching) depends on the oxygen diffusion rate into the polymer^37,38^. Such changes in the physicochemical properties of the polymer can strongly influence the leaching of phthalates from PVC microplastics^27,32^, and they should be taken into account when using leaching kinetics for environmental exposure assessment. The influence of UV irradiation on the leaching of phthalates has been investigated from high and low-density PE into aqueous solutions^39,40^ and from PVC to petroleum oils^41^. The leaching of phthalates from photoaged PVC microplastics into aqueous solutions has been investigated in only a few studies. These have demonstrated that photoaging can result in either higher or lower leaching rates. The experimental set-up (e.g., wet versus dry photoaging) influenced the amount of phthalates leached. Higher leaching rates were associated with changes in the chemical properties of the photoaged PVC, while lower leaching rates were associated with the degradation of phthalates to products not quantified in these studies^42–44^. The leaching process of phthalates from PVC impacted by UV light into water has received little attention and remains largely unknown.

Besides affecting the polymer structure, photoaging can lead to the transformation of additives contained in PVC, including plasticizers like phthalates^45^. Absorption of short-wave UV light and hydroxyl radical attack induces the transformation of DEHP to mono(2-ethylhexyl) phthalate (MEHP) and 2-ethylhexanol (Fig. S6). Further irradiation leads to the transformation of MEHP to phthalic acid. The formation of phthalic anhydride has been suggested but not confirmed^45^. The aforementioned transformation products have significantly different chemical properties compared to DEHP^46^, potentially altering the leaching process: they are smaller, more soluble in water and less hydrophobic^47^. These transformation products can also cause health problems as MEHP is suspected to interact with the hormone system in ways similar to DEHP, causing negative developmental and reproductive effects^48,49^. MEHP and phthalic acid can be toxic to aquatic invertebrates^50^. However, the leaching process of these transformation products from PVC microplastics has not yet been studied, and hence, there exists a major knowledge gap for exposure assessment of phthalates in aquatic systems.

In this study, we investigated the leaching of DEHP (which was used as a model phthalate) and its most abundant transformation products MEHP, phthalic acid, and phthalic anhydride from PVC microplastics into aqueous solutions. PVC microplastics were artificially photoaged under UV light for 24 and 48 days. We determined time-dependent leaching curves for DEHP from pristine and photoaged microplastics and, for the first time, also for transformation products. We identified the governing leaching process for each compound and elucidated the effects of photoaging on phthalates leaching.

## Results and discussion

### Photoaging enhanced the leaching of DEHP from PVC microplastics

The influence of photoaging on the leaching of DEHP and its transformation products was studied using spherical PVC microplastics with a radius of 2 mm, a surface area of 169 m^2^ g^−^^1^ and an initial DEHP content of approximately 38 wt%^27^. These model microplastics were artificially photoaged (dry aging) for 24 days and for 48 days under standardized laboratory conditions in a UV chamber^51^. PVC microplastics are denoted according to the degree of photoaging: PVC_Pristine_ (pristine PVC microplastics), PVC_24d_, and PVC_48d_ (PVC microplastics aged for 24 days and 48 days, respectively). The physicochemical properties of the PVC microplastics were determined before and after photoaging. Details on the characterization of PVC microplastics and photoaging conditions are provided at the beginning of the “Methods” section and in Supplementary Information S2. Laboratory batch-leaching experiments were conducted using pristine and photoaged PVC microplastics added to a 1 mM KCl solution using an infinite sink method^52^. At defined time intervals (after 1, 3, 5, 9, 16, 30, 50 and 80 days), the mass of DEHP and of each transformation product leached from the PVC microplastics was determined.

**Fig. 1:**
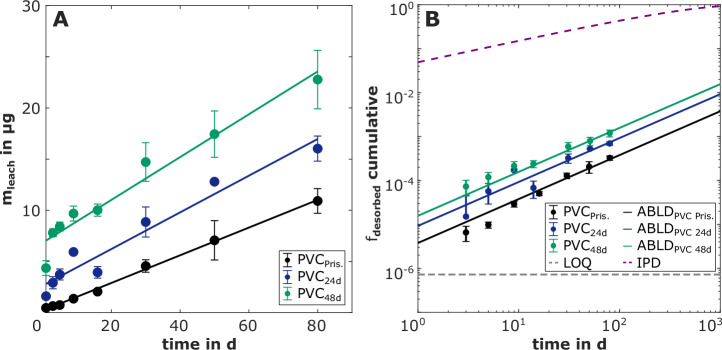
Leaching of DEHP from pristine and photoaged PVC microplastics. **A** Experimentally determined time-dependent leaching curves. The mass of DEHP leached (m_leach_ in µg, *y*-axis) from PVC_Pristine_^27^ (black circles), PVC_24d_ (blue circles) and PVC_48d_ (green circles) versus the respective sampling time (days, *x*-axis) are shown. The error bars represent one standard deviation (*n* = 3) calculated using Gaussian error propagation. The leaching curves can be described by linear regression lines (*R*^2^ = 0.947–0.998). **B** Fitting of the experimental data using mass transfer models for ABLD and IPD. The fitted ABLD model (solid lines in the respective color of the experiment) and the IPD model (dashed purple line) using the diffusion coefficient of DEHP in PVC D_PVC_ = 10^−^^14^ m^2^ s^−^^1^ (reported for PVC with comparable content of DEHP^54^) are shown. F_desorbed_ (*y*-axis) is the cumulative fraction of DEHP leached from the PVC microplastics. The limit of quantification (LOQ) of the method is indicated (dashed gray line).

Exposure of PVC microplastics to UV irradiation strongly increased the leaching of DEHP, therefore, leaching processes in the environment will be substantially faster than estimates based on pristine PVC microplastics. The time-dependent leaching curves of DEHP were linear for PVC_Pristine_ (*R*^2^ = 0.998, *p* = 4.78 × 10^−^^9^), PVC_24d_ (*R*^2^ = 0.947, *p* = 4.82 × 10^−^^5^), and PVC_48d_ (*R*^2^ = 0.953, *p* = 3.32 × 10^−^^5^) during 80 days of the experiment (Table S4.1). The mass of DEHP released instantaneously increased with the degree of photoaging of the PVC microplastics from 0.199 µg for PVC_Pristine_ to 2.64 µg and 6.83 µg for PVC_24d_ and PVC_48d_, respectively (Fig. 1). Continuous leaching rates (slopes of the time-dependent leaching curves) increased with the degree of photoaging from 0.135 µg d^−^^1^ for pristine PVC microplastics to 0.179 µg d^−^^1^ and 0.209 µg d^−^^1^ for PVC_24d_ and PVC_48d_, respectively.

Photoaging led to a 15% increase in surface area from 169 ± 3.18 m^2^ g^−^^1^ for PVC_Pristine_ to 195 ± 1.39 m^2^ g^−^^1^ for PVC_48d_ due to chain scission followed by crack formation, and thereby, to an increase in continuous and instantaneous leaching of DEHP^27^. The surface area of PVC_24d_ could not be determined using BET analysis because, in contrast to PVC_Pristine_ and PVC_48d_, PVC_24d_ microplastics were sticky, causing interferences during the measurements. The leaching rate increased by a factor of 1.3 and 1.5 from PVC_Pristine_ to PVC_24d_ and from PVC_Pristine_ to PVC_48d_, respectively. An increase in the leaching rate was also reported by Yan et al. ^42^ for the leaching of di-*n*-butyl phthalate (DnBP) from naturally photoaged PVC with a similar phthalate content (30 wt%) compared to the PVC microplastics used in this study^42^. Depending on the density of PE, UV irradiation during the experiments led to a 1.3 to 7.6-fold increase in the leaching rate of DEHP^40^. Exposure to artificial light led to a doubling of the released mass of dimethyl phthalate (DMP) and diethyl phthalate (DEP) from a PVC cable compared to the dark control^44^. In contrast, for PVC foil exposed to UVA light (320–400 nm)^53^ lower leaching rates of DEHP compared to the dark control were reported^43^. Less pronounced effects of photoaging could be due to the black color of the investigated PVC foil absorbing light and inhibiting the effect of photoaging into deeper layers or due to the phototransformation of DEHP during the experiment^38^.

### Leaching of DEHP from photoaged PVC microplastics is aqueous boundary layer diffusion limited

Phototransformation led to a decrease in the DEHP content of the PVC microplastics from 37.8 wt% for PVC_Pristine_^27^ to 22.6 wt% for PVC_24d_ and to 15.7 wt% for PVC_48d_ (*p* = 3.66 × 10^−^^6^, Table 1 and Table S2.4). The influence of thermal aging on the spatial distribution of DEHP was ruled out by time-of-flight secondary ion mass spectrometry (TOF-SIMS) analyses of the cross sections of PVC_Pristine_, PVC_24d_, and PVC_48d_. The analyses showed that the DEHP concentrations within the respective PVC microplastics (edge, middle, center) remained similar (Fig. S2.5). The results of the TOF-SIMS analysis confirmed the findings of the DEHP content measurements: The DEHP concentration was significantly different between the three PVC microplastics (*p* = 4.05 × 10^−^^8^, Table S2.5) and decreased from PVC_Pristine_ to PVC_24d_, and from PVC_24d_ to PVC_48d_. The reduction in the DEHP concentrations in PVC_24d_ and PVC_48d_ compared to PVC_Pristine_ suggested that phototransformation of DEHP had occurred throughout the PVC microplastics, from the surface to the core. With decreasing DEHP content, PVC microplastics became less flexible and D_PVC_ decreased^54^. D_PVC_ decreased from 8.45 × 10^−^^14^ m^2^ s^−^^1^ for PVC_Pristine_ to 4.58 × 10^−^^15^ m^2^ s^−^^1^ and 1.22 × 10^−^^15^ m^2^ s^−^^1^ for PVC_24d_ and PVC_48d_, respectively (Eq. 3, “Methods”). The calculations of D_PVC_ do not account for the effects of photoaging on the polymer structure. The surface of the polymer oxidized with increasing photoaging as indicated by an increase in the carbonyl index I_CO_ from 11.9 ± 0.46 for PVC_Pristine_ to 23.6 ± 1.34 for PVC_24d_ and to 50.6 ± 1.22 for PVC_48d_ (Eq. 1)^55^. While I_CO_ values close to zero have been reported for unaged PVC^56^, pristine PVC used in this study has a higher I_CO_ because of the high content of C=O-containing DEHP^42^. Photoaging led to the discoloration (yellowing) of the PVC microplastics (Fig. S2.2.1). Besides the photo-oxidized surface, a brownish subsurface layer indicating high polyene concentrations^37^ was identified from the cross section of the PVC microplastics (Fig. S2.2.2). UV light absorption in this layer hindered photochemical reactions in deeper layers, and the PVC microplastics became less yellow towards the center^38^. The glass transition temperature T_g_ increased from −33 °C for PVC_Pristine_ to −20 °C and −8 °C for PVC_24d_ and PVC_48d_, respectively, confirming the increasing degree of crosslinking in the polymer with prolonged exposure time to UV irradiation^57^. T_g_ of unplasticized PVC and DEHP are 80 °C and −83.5 °C, respectively. Higher T_g_ for PVC_24d_ and PVC_48d_ compared to PVC_Pristine_ result from the lower DEHP content in the polymer due to phototransformation^58,59^. A reduction in the polymer chain length due to chain scission results in a lower number-averaged molecular weight (M_n_) of the PVC microplastics^37^. With increasing exposure time to UV irradiation, M_n_ decreased from 76.2 kg mol^−^^1^ for PVC_Pristine_ to 7.04 kg mol^−^^1^ and 6.54 kg mol^−^^1^ for PVC_24d_ and PVC_48d_, respectively. Crosslinking reactions of polymer chains due to photoaging resulted in a higher weight-averaged molecular weight (M_w_) in PVC_24d_ (M_w_ = 260 kg mol^−^^1^) and PVC_48d_ (M_w_ = 237 kg mol^−^^1^) compared to PVC_Pristine_ (M_w_ = 182 kg mol^−^^1^). The lower solubilities in tetrahydrofuran (THF) of PVC exposed to UV light for more than 1000 h (equivalent to 42 days) could have resulted in a lower M_w_ of PVC_48d_ compared to PVC_24d_^57^. In the literature, the lowest M_w_ were found at the surface and in deeper layers of photoaged PVC, while the highest M_w_ values were found in polyene-rich subsurface layers^37^. A brownish subsurface indicates the polyene-rich layers^37^ in PVC_48d_ (Fig. S2.2.2).

**Table 1:** Mass of DEHP and its transformation products in ~85 mg of the respective PVC microplastics before the leaching experiments

	PVC_Pristine_	PVC_24d_	PVC_48d_
DEHP (µg)	32.1 × 10^3^ ± 937	19.2 × 10^3^ ± 359	13.3 × 10^3^ ± 495
MEHP (µg)	n.d.	506 ± 23.3	1.3 × 10^3^ ± 50.8
Phthalic acid (µg)	n.d.	11.0 ± 1.91	142 ± 8.01
Phthalic anhydride (µg)	n.d.	0.287 ± 0.0142	1.34 ± 0.0477

The standard deviations (*n* = 3) are provided.

*n.d.* not detected.

Photoaging-induced changes in the polymer structure of the PVC microplastics can lead to spatial variability of D_PVC_. Whereas D_PVC_ could be higher in the oxidized surface layer characterized by chain scission, crosslinking reduces diffusion processes and D_PVC_ in the core of the PVC microplastic could be lower than the calculated D_PVC_^41^. For IPD-limited leaching processes, lower D_PVC_ values slow down leaching (Eq. 2). The introduction of oxygen-containing functional groups with increasing exposure time to UV light (Fig. S2.3.2) increased the surface polarity and hydrophilicity of the PVC microplastics and led to lower partition coefficients, K_PVC/W_^39,42,44^. Decreasing K_PVC/W_ values accelerate the leaching of DEHP for ABLD-limited leaching processes (Eq. 4)^27,42^.

While the leaching of DEHP from pristine PVC microplastics is ABLD-limited^27^, we investigated if photoaging led to a shift of the limiting mass-transfer process from ABLD to IPD. This shift in mass transfer would substantially change the leaching kinetics of DEHP into aqueous systems and its susceptibility to effects from environmental factors^31^. However, IPD could not describe the experimental data and ABLD was clearly the governing diffusion process for the leaching of DEHP from photoaged PVC (Fig. 1). The log K_PVC/W_ decreased from 8.55 for PVC_Pristine_ to 8.21 and 7.98 for PVC_24d_ and PVC_48d_, respectively, and was the decisive parameter for the increase in both continuous and instantaneous leaching of DEHP from photoaged PVC microplastics. The reduction in K_PVC/W_ can be attributed to the introduction of oxygen-containing functional groups, such as C=O (Fig. S2.3.3), and the decreased concentration of DEHP in photoaged PVC microplastics (c_PVC_). This resulted in a more pronounced change in K_PVC/W_ in comparison to the leaching rates. Lower K_Polymer/W_ have been observed for DEHP and DnBP after exposure of PE and PVC to UV light and to sunlight, respectively^40,42^.

In well-mixed laboratory batch experiments similar to this study, the leaching of hydrophobic contaminants, namely tonalide (log K_O/W_ = 5.7)^23^ and polychlorinated biphenyls (log K_O/W_ = 4.90–8.19)^24^ from spherical PE microplastics was also limited by ABLD with the partition coefficient K_PE/W_ being the decisive parameter. The release of brominated flame retardants (log K_ABS/W_ = 2–3.1) from acrylonitrile butadiene styrene (ABS) was limited by IPD due to the lower partition coefficients^25^. The leaching of less hydrophobic hexachlorocyclohexanes (log K_O/W_ ≤ 4.14) from PE and PP films was IPD-limited, while the leaching of chlorinated benzenes with higher partition coefficients (log K_O/W_ ≥ 5.17) was ABLD-limited^26^.

Leaching half-lives of DEHP from pristine and photoaged PVC microplastics into water were calculated using the aqueous diffusion coefficient D_aq_, the aqueous boundary layer thickness δ and the respective K_PVC/W_ (Eq. 5)^27^. The corresponding leaching half-lives were 449 years for the leaching of DEHP from PVC_Pristine_, 205 years from PVC_24d_ and 121 years from PVC_48d_. Compared to the influence of other environmental factors, photoaging had a more pronounced effect on the leaching half-lives of DEHP from PVC microplastics than ionic strength and water temperature but a less pronounced effect than flow conditions, dissolved organic carbon, and fragmentation (particle size)^27,31^. In aquatic environments, these factors are subject to a high spatial-temporal variability, and their interaction must be taken into account when studying leaching processes.

### Photoaging enhanced the leaching of transformation products from PVC microplastics

**Fig. 2:**
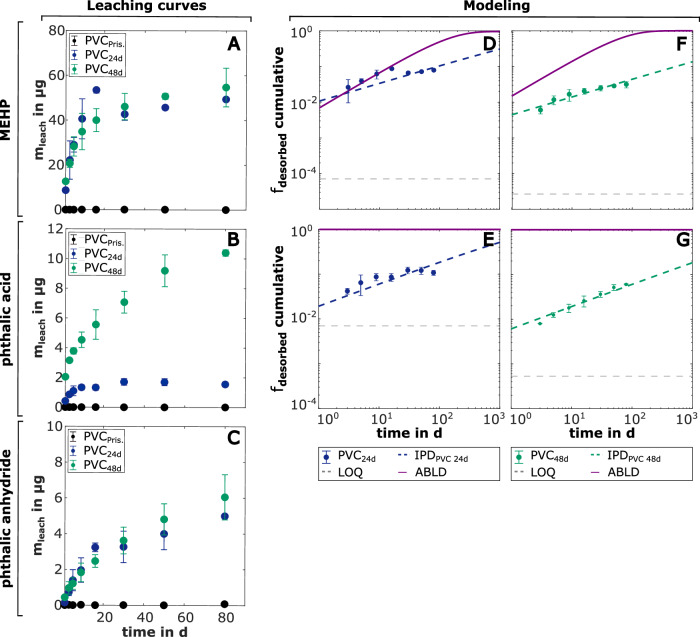
Leaching of the transformation products from photoaged PVC microplastics. **A**–**C** Time-dependent leaching curves of the transformation products. The mass of MEHP, phthalic acid, and phthalic anhydride leached from PVC_Pristine_ (black circles), PVC_24d_ (blue circles) and PVC_48d_ (green circles) (m_leach_ in µg, *y*-axis) versus the respective sampling time (days, *x*-axis) are shown. The error bars represent one standard deviation (*n* = 3) calculated using Gaussian error propagation. **D**–**G** Fitting of the experimental data using mass transfer models for ABLD and IPD. The ABLD model (solid purple line) and the IPD model (dashed lines in the respective color of the experiment) for MEHP and phthalic acid are shown. F_desorbed_ (*y*-axis) is the cumulative fraction of each transformation product leached from the PVC microplastics. For the leaching of MEHP from PVC_48d_ and for phthalic acid, K_O/W_ of the compound was used to calculate the ABLD model. The limit of quantification (LOQ) of the method is indicated (dashed gray line).

Photoaging of PVC microplastics led to higher leaching rates of MEHP, phthalic acid, and phthalic anhydride. PVC_Pristine_ did not contain any of these transformation products, and they were not observed in the time-dependent leaching curves (Table 1, Fig. 2). Slightly more MEHP leached from PVC_48d_ compared to PVC_24d_, due to the higher initial MEHP content (*p* = 2.95 × 10^−^^5^, Table S2.4) in PVC_48d_. Leaching rates of MEHP from PVC_24d_ and PVC_48d_ were higher for the first 16 days than for later time points (Table 2, Table S4.2). After 80 days, 49.4 µg (10% of the initial content) and 54.7 µg (4% of the initial content) of MEHP were released from PVC_24d_ and PVC_48d_, respectively, indicating that MEHP depletion was not reached. In comparison, 16.0 µg (PVC_24d_) and 22.8 µg (PVC_48d_) of DEHP, corresponding to 0.08% and 0.17% of the initial content, respectively, were leached after 80 days. MEHP was leached faster than DEHP because (1) MEHP is less hydrophobic (K_PVC/W_ is lower) and diffuses faster through the ABL, and (2) MEHP is smaller and diffuses faster through PVC^46^.

**Table 2:** Leaching rates (and standard errors) of transformation products from PVC_24d_ and PVC_48d_

	PVC_24d_		PVC_48d_	
	1–16 d	16–80 d	1–16 d	16–80 d
MEHP (µg d^−^^1^)	2.03 ± 0.638	0.125 ± 0.008	1.72 ± 0.385	0.221 ± 0.0415
Phthalic acid (ng d^−^^1^)	53.1 ± 21.2	2.24 ± 4.21	223 ± 34.2	74.6 ± 12.2
Phthalic anhydride (ng d^−^^1^)	199 ± 16.4	29.2 ± 4.15	130 ± 14.0	54.9 ± 5.92

Photoaging led to a 13-fold increase in the phthalic acid content (*p* = 2.34 × 10^−^^5^, Table 1 and Table S2.4) in PVC_48d_ compared to PVC_24d_, consequently leading to higher leaching quantities. For instance, after 80 days, 10.4 µg of phthalic acid was leached from PVC_48d_ compared to 1.56 µg from PVC_24d_. As for MEHP, the leaching rates of phthalic acid were higher for the initial 16 days than for subsequent time points (Table 2, Table S4.2).

The amount of phthalic anhydride leached after 80 days increased from 4.99 µg for PVC_24d_ to 6.05 µg for PVC_48d_ due to the higher initial concentration in PVC_48d_ compared to PVC_24d_ (*p* = 7.35 × 10^−^^6^, Table 1 and Table S2.4). The time-dependent leaching curves had a steeper slope at the beginning of the experiment and a flatter slope at later leaching times (Table 2, Table S4.2). The initial content of phthalic anhydride in photoaged PVC microplastics was lower than the mass leached, probably due to the reaction of phthalic anhydride with THF during the dissolution of the PVC microplastics (the dissolution was required to determine the phthalic anhydride content in the microplastics, S2.4) resulting in the formation of products not quantified in this study^60^.

### Intraparticle diffusion governs the leaching of transformation products

At the beginning of the leaching experiment (1–16 days), ABLD was the rate-limiting process for the leaching of MEHP from PVC_24d_ (log K_PVC/W_ = 5.40) (Fig. 2). The IPD model could not adequately fit the experimental data due to relatively high D_PVC_ and thus, fast diffusion of MEHP in the oxidized surface layer of the photoaged PVC microplastics characterized by chain scission. After surface depletion, the diffusion of MEHP slowed down due to lower D_PVC_ in the cross-linked deeper layers of the PVC microplastics. A shift in mass transfer could be observed, and IPD became the limiting mass transfer process for the leaching of MEHP from PVC_24d_ at later time points; D_PVC_ was 5 × 10^−^^16^ m^2^ s^−^^1^. For PVC_48d_, introducing oxygen-containing functional groups with proceeding photoaging increased the surface hydrophilicity of the PVC microplastics leading to lower K_PVC/W_ of MEHP for PVC_48d_ than for PVC_24d_. Thereby, ABLD became faster and could not describe the experimental data. IPD was the rate-limiting mass transfer process for the leaching of MEHP from PVC_48d_. D_PVC_ was lower for PVC_48d_ (D_PVC_ = 8 × 10^−^^17^ m^2^ s^−^^1^) than for PVC_24d_ because of increasing crosslinking of the polymer with increasing exposure time to UV light, slowing down IPD. Leaching of MEHP from PVC_48d_ was faster at the beginning of the experiments due to higher D_PVC_ in the surface layer. With increasing leaching time, leaching slowed down, since diffusion of MEHP in deeper layers of the photoaged PVC microplastics was slower. Fitted D_PVC_ values for IPD represent an average value for the PVC microplastics. Compared to chain scission, crosslinking reactions lead to heterogeneous polymer structures with local accumulation of conjugated polyenes^37,38^. In the aquatic environment, floating plastic debris are mostly not evenly photoaged^61^. To better describe IPD-limited leaching from photoaged PVC microplastics, numerical models accounting for the spatial variability of D_PVC_ require consideration in future research^54^. For ABLD-limited leaching, heterogeneous photoaging of the surface of the microplastics resulting in spatial variability of K_PVC/W_ must be taken into account. Photoaging-induced changes in the concentration of the diffusing compound altering K_PVC/W_ also need to be integrated into numerical models.

The ABLD model did not fit the time-dependent leaching curves for phthalic acid from PVC_24d_ and PVC_48d_ because phthalic acid is rather hydrophilic (log K_O/W_ = 0.74). IPD was also the rate-limiting process for phthalic acid from photoaged PVC microplastics (Fig. 2). IPD slowed down with proceeding leaching time due to higher D_PVC_ at the surface and lower D_PVC_ values in deeper layers of the PVC microplastics. D_PVC_ decreased from 1.5 × 10^−^^16^ m^2^ s^−^^1^ for PVC_24d_ to 1.5 × 10^−^^17^ m^2^ s^−^^1^ for PVC_48d_ because crosslinking reactions in the polymer increased with exposure time to UV light.

Time-dependent leaching curves for phthalic anhydride were not evaluated using the ABLD and IPD models because the initial content in the photoaged PVC microplastics could not be determined. Phthalic anhydride is more hydrophilic than phthalic acid (Table 3), and ABLD will be relatively fast compared to IPD. Due to similar molecular weight and structure, similar D_PVC_ can be assumed for phthalic acid and phthalic anhydride. IPD is expected to be the governing diffusion process for the leaching of phthalic anhydride from photoaged PVC microplastics.

**Table 3:** Selected chemical properties of DEHP and its transformation products

	DEHP	MEHP	Phthalic acid	Phthalic anhydride
Mol. weight (g mol^−^^1^)^a^	390.6	278.3	166.1	148.1
Log K_O/W_ (at 20 °C)^a^	7.66	5.08	0.74	−0.71
Water solubility (mg L^−^^1^ at 20 °C)^a^	3.6 × 10^−^^3^	2.9	533	2.45 × 10^4^
Boiling point (°C)^a^	457.2	406.7	366.4	277.0
D_aq_ (× 10^−^^10^ m^2^ s^−^^1^)^b^	4.45	5.33	7.00	7.44

^a^SPARC calculator^47^.

^b^Calculated^70^.

## Conclusions

Photoaging strongly enhances the leaching of DEHP from PVC microplastics and leads to the formation and enhanced release of transformation products into aquatic environments. Leaching of DEHP from pristine and photoaged microplastics is governed by ABLD due to high K_PVC/W_. Leaching of transformation products from photoaged microplastics is in most cases governed by IPD because K_PVC/W_ is low and photoaging leads to crosslinking in the polymer and, thereby, to lower D_PVC_.

This study provides a mechanistic understanding of the effects of photoaging on the leaching of phthalates and transformation products from PVC microplastics into aquatic systems. The solar UV radiation in the environment exhibits considerable natural variability. Diurnal and annual variations, as well as the latitude, altitude, and weather conditions (e.g., UV attenuation by clouds), strongly impact UV radiation^62^. Site-specific parameters must be taken into account when investigating the photoaging phenomena of plastics in the environment. In addition, leaching processes in complex aquatic environments can be significantly influenced by a number of environmental factors, including flow conditions, water chemistry, mechanical stress, and microbial activity (e.g., biofilm formation), resulting in altered leaching times such as half-lives^27^. The leaching models and calculations presented here can be adapted to the specific environmental conditions.

While environmental processes can facilitate the degradation of harmful additives, transformation products (such as monoesters in the case of phthalates) can cause a number of adverse health effects, too^63^. To assess the chemical pollution of the environment by additives emitted from plastics, it is not only necessary to understand the leaching process but to consider the transformation of additives and to account for the effect of environmental factors that have a decisive influence on leaching kinetics and processes. Both aspects must be factored in for a well-founded prediction of the risk of (micro)plastics, otherwise the consequences of exposure will be underestimated.

The release of ortho-phthalates such as DEHP from PVC microplastics has only recently been recognized as a risk to humans and the environment. Our results highlight the risk of long-term release of harmful phthalates and transformation products from PVC microplastics into the aquatic environment, and the identified mass transfer rates can contribute to the adaption of regulations and enforcing restrictions^21,64^. Ortho-phthalates have been used for decades in various everyday plastic applications, resulting in decades of exposure to aquatic life and humans. In terms of sustainable chemical management, our findings support the need to minimize the release of additives into the environment^21^. In addition, strategies to reduce the environmental footprint of plastic additives would need to include a reduction of plastic production (as plastics are the intended use of additives), the recovery and recycling of additives, the use of sustainable resources and renewable energy in the additive production, and the replacement of harmful additives with benign and environmentally friendly alternatives that are not harmful at any stage of their life cycle.

## Methods

### Dry UV-aging of PVC microplastics

Information on all used chemicals and instruments is provided in Supplementary Information S1. In this study, model PVC microplastics purchased from Industrie Generali spa (Samarate, Italy) were used to investigate the influence of photoaging on phthalates leaching. The PVC microplastics (28 ± 0.5 mg per pellet) were spherical with a radius of 2 mm and contained 37.8 wt% of DEHP (C_24_H_38_O_4_)^65^ determined in the laboratory in our previous study^27^. DEHP was the only plasticizer identified in the PVC microplastics. Plastic particles detected in the environment can differ from the microplastics used in this study, e.g., in size and shape^66^. Calculations on the mass transfer processes need to be adjusted accordingly^67^. In addition, the penetration depth of UV light might vary for particles of different shapes and sizes and requires consideration^61^. PVC microplastics were artificially photoaged under controlled laboratory conditions according to DIN EN ISO 4892-2^51^. Therefore, a Suntest CPS+ photoaging chamber equipped with a NXe 1500 B xenon lamp (both: Atlas/AMETEK, Linsengericht, Germany) was used. PVC microplastics were placed in two quartz glass Petri dishes, covered with a lid, and placed in the UV chamber for 24 or 48 days. The distance between the pellets was ~0.3 cm. In the UV chamber, PVC microplastics were exposed to a UV spectrum of 300–400 nm, representing the UV radiation of terrestrial systems^53^. The UV intensity was 65 W m^−^^2^_._ The black standard temperature (BST) and the temperature in the chamber were 65 °C and 40 ± 2 °C, respectively. To ensure microplastics were exposed evenly to UV irradiation in the chamber, Petri dishes were rotated 90° clockwise every 3 days, and the PVC microplastics were turned upside down after half of the total aging time. Regular opening of the Petri dishes ensured sufficient oxygen supply for photooxidation reactions. The accumulated UV irradiation was 135 MJ m^−^^2^ after 24 days and 269 MJ m^−^^2^ after 48 days. For ambient Central European conditions, about 216 MJ m^−^^2^ per year can be assumed^68^. This means that 24 days and 48 days of artificial photoaging correspond to 7.5 and 15 months of aging in the environment. PVC microplastics are denoted according to the degree of photoaging: pristine PVC microplastics are denoted PVC_Pristine_, PVC microplastics after 24 days and 48 days of photoaging are denoted PVC_24d_ and PVC_48d_, respectively.

PVC microplastics were thoroughly characterized before and after photoaging to identify the effect of photoaging-induced changes of the polymer on the leaching of DEHP and transformation products. Selected chemical properties of DEHP and its transformation products MEHP (C_16_H_22_O_4_)^65^, phthalic acid (C_8_H_6_O_4_)^65^, and phthalic anhydride (C_8_H_4_O_3_)^65^ are given in Table 3. Details on the respective measurement and characterization of the microplastics are provided in Supplementary Information S2. PVC microplastics were weighed before and after aging. Surface functional groups were determined using Attenuated Total Reflectance-Fourier Transform Infrared Spectroscopy (ATR-FTIR). The content of DEHP and of the transformation products of pristine and photoaged PVC microplastics was determined following the standard and adapted operation procedure for consumer product safety^69^. The spatial distribution of DEHP in pristine and photoaged PVC microplastics was determined using time-of-flight secondary ion mass spectrometry (TOF-SIMS) analysis. The surface area of the PVC microplastics was determined using Brunauer-Emmett-Teller (BET) analysis. The molar mass distribution of the PVC microplastics was measured using Gel Permeation Chromatography (GPC), and the glass transition temperature was determined using Differential Scanning Calorimetry (DSC).

To assess the degree of photoaging of the PVC microplastics, the carbonyl index (I_CO_) was calculated. I_CO_ describes the ratio between the absorbance of carbonyl groups to the absorbance of a reference group, e.g., the methylene bond and can be determined from ATR-FTIR spectra. I_CO_ can be expressed as follows^55^:

1 $${{{{\rm{I}}}}}_{{{{\rm{CO}}}}}=\frac{{{{{\rm{A}}}}}_{{{{\rm{C}}}}={{{\rm{O}}}}}}{{{{{\rm{A}}}}}_{{{{\rm{ref}}}}}}$$

where A_C=O_ and A_ref_ are the peak area of the carbonyl bond and the reference bond, respectively. For A_C=O_, the integrated signal at 1610–1830 cm^−^^1^ was used. The peak area 1440–1480 cm^−^^1^ corresponding to C-H groups was chosen for A_ref_^56^. Four to five spectra were evaluated to calculate the mean I_CO_ for each PVC microplastics (Fig. S2.3.1).

### Leaching experiments

All glassware was rinsed with ultra-pure water, then with acetone, and heated at 550 °C for 5 h in a muffle oven. Closures were equipped with a Teflon septa. All experiments were conducted using three sample replicates and six blanks (consisting of an infinite sink and the background solution). For the leaching experiments, an infinite sink method was applied. Details on the infinite sink method for phthalate analysis are described elsewhere^52^. For investigating the leaching of the more hydrophilic transformation products (Table 1), the method was modified (Fig. S1). Briefly, 10 mg of activated carbon was weighed on a piece of filter paper, folded, and the sink was stabilized by a 0.35 mm stainless steel wire. The sinks were equilibrated in 40 mL of 1 mM KCl solution at neutral pH (pH = 6.69 ± 0.16) on a horizontal shaker at 125 rpm overnight. To focus on the effects of photoaging on leaching, potential interference from biotic weathering was excluded by adding sodium azide (50 mg L^−^^1^) to the aqueous solutions. 85 ± 0.5 mg of PVC microplastics were added, and the vials were placed on a horizontal shaker at 20 °C until sampling. To avoid photodegradation during the leaching experiments, samples were kept in the dark. At sampling intervals of 1–80 days, first the PVC microplastics and then the infinite sink were taken out with tweezers. For the quantification of DEHP, the infinite sinks were spiked with 1 µg of deuterated internal DEHP-d_4_ standard. To quantify the transformation products MEHP, phthalic acid and phthalic anhydride, the infinite sinks were additionally spiked with 1 µg of deuterated internal phthalic acid-d_4_ standard. Sinks were dried at 40 °C and stored in a desiccator until extraction.

DEHP was extracted from the infinite sinks using an accelerated solvent extractor (ASE) at 120 °C using n-hexane as solvent. The transformation products were afterward extracted at 100 °C using methanol as solvent. For the quantification of transformation products in the water phase, 5 mL were spiked with 1 µg of phthalic acid-d_4,_ and 1 mL was filtered with a 0.45 µm cellulose acetate filter in a 1.5 mL brown glass measurement vial. The methanol extracts from the solid-phase extraction were concentrated to 500 µL. The transformation products in the methanol extracts (m_sink_) as well as in the water phase (m_w_) were quantified using a liquid chromatograph coupled to a triple-quadrupole mass spectrometer (LC-MS/MS) (S1). For the quantification of DEHP in water, 20 mL of the aqueous solution was spiked with 1 µg of DEHP-d_4_ and extracted using liquid-liquid extraction with three times 5 mL of n-hexane. The extracts were pooled in a 20 mL vial. The hexane extracts were concentrated to 100 µL, and the amount of DEHP in the infinite sink (m_sink_) and in the water phase (m_w_) were quantified using a gas chromatograph coupled to a triple-quadrupole mass spectrometer (GC-MS/MS) (S1). By conducting the leaching experiments, the mass of DEHP, MEHP, phthalic acid, and phthalic anhydride leached from the PVC microplastics m_leach_ (= m_sink_ + m_w_) was determined. By plotting m_leach_ versus the respective sampling time, time-dependent leaching curves for each compound were obtained. The time-dependent leaching curve of DEHP from PVC_Pristine_ has been determined in an earlier study^27^.

A recovery test was conducted by spiking each 2.5 µg of DEHP, MEHP, phthalic acid, and phthalic anhydride into 40 mL of a 1 mL KCl solution containing the infinite sink. The vials were placed on the horizontal shaker at 125 rpm for 5 days. The experiments were then carried out as described above. The recovery test was conducted using triplicates. The recoveries were 95 ± 9% for DEHP, 98 ± 12% for MEHP, 70 ± 3% for phthalic acid and 92 ± 11% for phthalic anhydride.

To ensure transformation products measured in the framework of the leaching experiments resulted from the photoaging of the PVC microplastics and not from further transformation processes during the leaching experiments, the stability of each compound in water was measured over 24 days using triplicates (S3). MEHP, phthalic acid and phthalic anhydride were stable throughout the experiment, with a recovery of 117 ± 6% for MEHP, 99.6 ± 2% for phthalic acid, and 100 ± 4% for phthalic anhydride (Fig. S3, Table S3).

### Leaching process

Phthalates leaching is composed of instantaneous and continuous leaching. Instantaneous leaching results from the diffusion of a compound and the formation of a layer on the surface of the PVC microplastics and, can be obtained from the y-intercept of the time-dependent leaching curves^27^. Continuous leaching can be determined from the slope of the leaching curves. To exclude the influence of instantaneous leaching for the evaluation of continuous leaching, the mass of DEHP leached instantaneously was subtracted from m_leach_. Since time-dependent leaching curves for the transformation products were not linear, the y-intercept could not be determined, and the mass leached after 1 day was subtracted from m_leach._

Continuous leaching of a compound from PVC microplastics consists of sequential internal and external diffusion processes. The slower of both processes governs the overall diffusion^28^. The internal diffusion process is called intraparticle diffusion (IPD) and describes the diffusion of a compound within the PVC microplastics. For this experimental set-up using spherical PVC microplastic particles, IPD can be expressed as follows^24^:

2 $${{{{\rm{f}}}}}_{{{{\rm{desorbed}}}}}=1-\frac{{{{{\rm{M}}}}}_{{{{\rm{t}}}}}}{{{{{\rm{M}}}}}_{0}}=1-\frac{6}{{{{{\rm{\pi }}}}}^{2}}{\sum}_{{{{\rm{n}}}}=1}^{\infty }\frac{1}{{{{{\rm{n}}}}}^{2}}{{{{\rm{e}}}}}^{\left(-{{{{\rm{n}}}}}^{2}{{{{\rm{\pi }}}}}^{2}\frac{{{{\rm{t}}}}{{{{\rm{D}}}}}_{{{{\rm{PVC}}}}}}{{{{{\rm{r}}}}}^{2}}\right)}$$

where f_desorbed_ (-) is the fraction of the compound desorbed at each time t (d), M_t_ (µg) and M_0_ (µg) are the mass of the compound in the PVC microplastics at t and the initial mass, respectively, and r is the radius (m) of the PVC microplastics. M_0_ for DEHP in PVC_Pristine_ was known^27^ and M_0_ of DEHP in photoaged PVC and of the transformation products was determined in this study. A Taylor number *n* = 1–10,000 was used to approximate the IPD model. D_PVC_ (m^2^ s^−^^1^) is the diffusion coefficient of the compound in PVC. Based on its relationship with the DEHP content (x) of the PVC microplastics, D_PVC_ was calculated by^30^:

3 $${{{{\rm{D}}}}}_{{{{\rm{PVC}}}}}={{{{\rm{D}}}}}_{{{{\rm{PVCzero}}}}} \times {{{{\rm{e}}}}}^{{{{\rm{ax}}}}}$$

where D_PVCzero_ (m^2^ s^−^^1^) is the diffusion coefficient of DEHP in PVC with a DEHP content of zero, and a (-) is the plasticization power, indicating the efficiency of DEHP. D_PVCzero_ is 6 × 10^−^^17^ m^2^ s^−^^1^ and a is 19.2^27^.

The external diffusion process is called aqueous boundary layer diffusion (ABLD) and describes the diffusion of a compound through an aqueous boundary layer on the surface of the PVC microplastics. For this experimental set-up using spherical PVC microplastics, ABLD can be expressed as follows^24^:

4 $${{{{\rm{f}}}}}_{{{{\rm{desorbed}}}}}=1-\frac{{{{{\rm{M}}}}}_{{{{\rm{t}}}}}}{{{{{\rm{M}}}}}_{0}}=1-{{{{\rm{e}}}}}^{\left(-\frac{{3{{{\rm{D}}}}}_{{{{\rm{aq}}}}}{{{\rm{t}}}}}{{{{\rm{r}}}}{{{\rm{\delta }}}}\,{{{{\rm{K}}}}}_{{{{\rm{PVC}}}}/{{{\rm{W}}}}}}\right)}$$

where D_aq_ (m^2^ s^−^^1^) is the aqueous diffusion coefficient of the compound, δ (m) is the ABL thickness and K_PVC/W_ (L L^−^^1^) is the partition coefficient of compound between PVC and water. K_PVC/W_ describes the ratio of the concentration of a compound in PVC (c_PVC_, µg L^−^^1^) to its concentration in water (c_w_, µg L^−^^1^). Using the density of the PVC microplastics, K_PVC/W_ can be converted to L kg^−^^1^.

Specific leaching times, such as leaching half-lives, can be calculated when the limiting mass transfer process is known. The leaching half-life expresses the time it takes until 50% of DEHP or of the transformation products have leached from the PVC microplastics. For ABLD-limited leaching, half-lives can be calculated as follows^24^:

5 $${{{{\rm{t}}}}}_{1/2}=\frac{{{{\rm{r}}}}\,{{{{\rm{\delta }}}}{{{\rm{K}}}}}_{{{{\rm{PVC}}}}/{{{\rm{W}}}}}{{\mathrm{ln}}}(2)}{3{{{{\rm{D}}}}}_{{{{\rm{aq}}}}}}$$

### Parameter fitting

The cumulative fraction of DEHP and each transformation product leached from the PVC microplastics over time was evaluated using IPD and ABLD models, and the governing diffusion process was identified. For IPD log D_PVC_ was fitted, and for ABLD log K_PVC/W_ was optimized. D_aq_ of DEHP (= 4.45 × 10^−^^10^ m^2^ s^−^^1^) and δ = 38.4 µm for this experimental set-up have been determined in an earlier study^27^. D_aq_ for MEHP, phthalic acid, and phthalic anhydride were calculated^70^. All calculations were made in MATLAB R2018a; the tool *fminsearch* was used for the fitting of the time-dependent leaching data. The sum of the squared differences between the experimental and model values for f_desorbed_ was chosen as an objective function.

## Supplementary information

Supplementary Information

## Data Availability

All data generated or analyzed during this study are included in this published article (and its Supplementary Information file).
