# Supplementary material for: Photoaging enhances the leaching of di(2-ethylhexyl) phthalate and transformation products from polyvinyl chloride microplastics into aquatic environments
Source: Commun Chem. 2024 Sep 27;7:218. doi: 10.1038/s42004-024-01310-3 (PMC11436666; doi:10.1038/s42004-024-01310-3)
Supplement: Supplementary file 1 — Supplementary Information [file 42004_2024_1310_MOESM1_ESM.pdf]

Supplementary Information to

Photoaging enhances the leaching of di(2-ethylhexyl) phthalate and transformation products from polyvinyl chloride microplastics into aquatic environments

Charlotte Henkel[†‡‖§], *Thorsten Hüffer*[†‖*], *Ruoting Peng*[†‡], *Xiaoyu Gao*[§], *Subhasis Ghoshal*[§], *Thilo Hofmann*[†‖*]

† University of Vienna, Centre for Microbiology and Environmental Systems Science, Department for Environmental Geosciences, Josef-Holaubek-Platz 2, 1090 Vienna, Austria.

‡ University of Vienna, Doctoral School in Microbiology and Environmental Science, Djerassiplatz 1, 1030 Vienna, Austria.

‖ University of Vienna, Research Platform Plastics in the Environment and Society (Plenty), Josef-Holaubek-Platz 2, 1090 Vienna, Austria.

[§] McGill University, Department of Civil Engineering, 817 Sherbrooke Street West, Montreal, Quebec H3A 0C3, Canada.

Corresponding Authors:

[*] Thilo Hofmann; Phone: +43-1-4277-53320; Email: thilo.hofmann@univie.ac.at

[*] Thorsten Hüffer; Phone: +43-1-4277-53383; Email: thorsten.hueffer@univie.ac.at

Communications Chemistry

Pages: 24

Figures: 11

Tables: 8

S1 Description of chemicals and instruments

Chemicals

Organic solvents including n-hexane (≥ 95 %, PESTINORM®), acetone (Anala® NORMAPUR) and 2-propanol (Anala® NORMAPUR) and methanol (LiChrosolv®) were purchased from VWR Chemicals (Vienna, Austria). Formic acid (98–100 %, HPLC LiChropur) and tetrahydrofuran (THF, ≥ 99 %) were purchased from Merck (Darmstadt, Germany). Ultra-pure water was obtained from a Purlab® Chorus 1 Complete water purification system (ELGA LabWater, Veolia Water Technologies, Celle, Germany). The infinite sink was composed of activated carbon powder Norit® SAE SUPER (Cabot Norit Nederland B.V., Klazienaveen, The Netherlands), Whatman Grade 50 filter paper (GE Heathcare, Dassel, Germany) and stainless steel (V4a 317L) wire of 0.35 mm thickness (Zivipf, Treuchlingen, Germany). Potassium chloride (Anala® NORMAPUR) was purchased from VWR Chemicals (Vienna, Austria). Sodium azide (99 %) was purchased from Thermo Fisher Scientific (Waltham, Massachusetts, U.S.). DEHP (Pestanal®, analytical standard), DEHP-d_4 (analytical standard), MEHP (97 %), phthalic acid (analytical standard), phthalic acid-d_4 (≥ 98 atom % D) and phthalic anhydride (ACS reagent, ≥ 99 %) were purchased from Merck (Darmstadt, Germany). For DEHP, stock standards (5000 µg mL^{-1}, 500 µg mL^{-1} and 50 µg mL^{-1}) and a DEHP-d_4 standard (50 µg mL^{-1}) in n-hexane were prepared in amber brown glass vials and stored at 4 °C in the dark. These were further used to prepare the calibration standards for the gas chromatography- triple quadrupole mass spectrometry (GC-MS/MS) measurements. For MEHP, phthalic acid, and phthalic anhydride a mixed stock standard (50 µg mL^{-1}) in methanol and for phthalic acid-d_4 a standard (50 µg mL^{-1}) in methanol were prepared in amber brown glass vials and stored at 4 °C

in the dark. These standards were used to prepare the calibration standards for the liquid chromatography- triple quadrupole mass spectrometry (LC-MS/MS) measurements. Additionally, a DEHP-d4 working standard (50 µg mL^{-1}) in 2-propanol and a phthalic acid-d4 working standard (50 µg mL^{-1}) in methanol were prepared for spiking the infinite sinks and the water phase.

Instruments

pH was measured using a Multi 9620 IDS multi-parameter benchtop meter (WTW, Weilheim, Germany). The balance was an AT201 (Metler Toledo GmbH, Vienna, Austria). For the solid phase extraction, an accelerated solvent extractor (ASE 350, Thermo Fisher Scientific, Waltham, U.S.) was used. Extracts were concentrated using a laboratory evaporator (Barkey vapotherm basic mobil I, Leopoldshöhe, Germany). Leaching experiments were conducted on a horizontal shaker (SM-30, shaker amplitude 46 mm, Edmund Bühler GmbH, Bodelshausen, Germany).

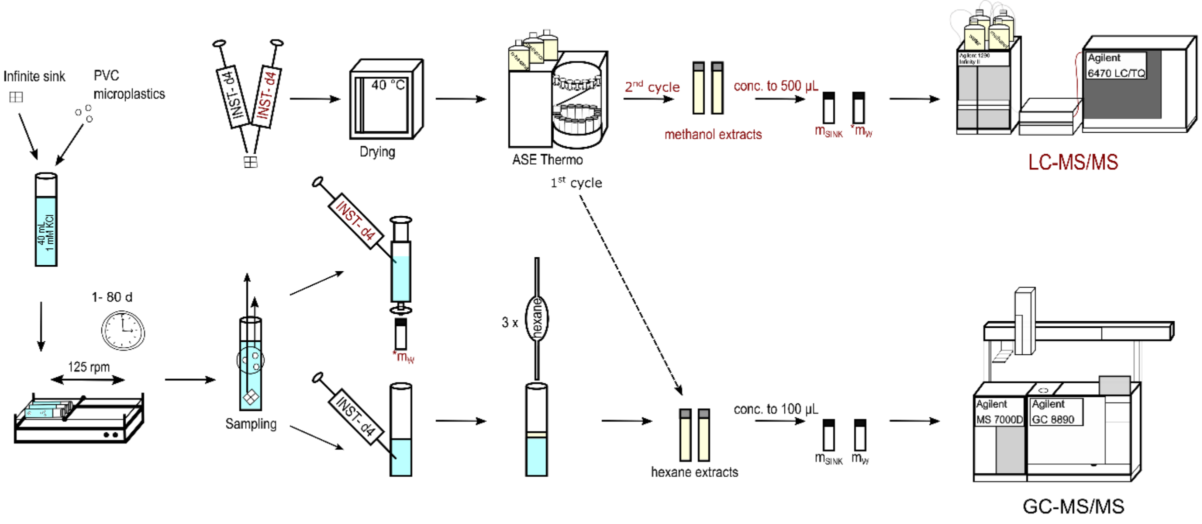

Figure S1. Experimental set-up for the quantification of DEHP and its transformation products leached from PVC microplastics using LC-MS/MS and GC-MS/MS.

Quantification of DEHP

DEHP was quantified using a GC 8890 coupled to a triple quadrupole MS 7000 (both Agilent Technologies, Santa Clara, U.S.) and a PAL 3 (RSI 120) autosampler (CTC Analytics AG, Zwingen, Switzerland). The injection volume was 1 µL. The MM Inlet mode was split (15:1) with an initial temperature of 70 °C followed by a ramp of 900 °C min^{-1} to 300 °C. Two HP-5ms ultra inert columns (15 m x 250 µm x 0.25 µm, Agilent Technologies, Santa Clara, U.S.) were used for the chromatographic separation and Helium 5.0 (Linde Gas GmbH, Stadl-Paura, Austria) served as carrier gas. The column flow was 1.4 mL min^{-1} for the first column and 1.5 mL min^{-1} for the second column. The oven temperature was 40 °C held for 2 min, followed by a ramp of 50 °C min^{-1} to 300 °C held for 4 min. The MS/MS parameters were as follows: the EI-source temperature was 230 °C, the electron energy was 70 eV. All transitions are given in **Table S1.1**.

Table S1.1. MS parameters for the quantification of DEHP. The transitions used as quantifiers are indicated by an asterisk.

**Compound**	**Precursor ion (m/z)**	**Product ion (m/z)**	**Collision energy (V)**	**Retention time (min)**
DEHP	279	149	16	8.6
DEHP	149	121	18	8.6
DEHP	149	93	21	8.6
DEHP*	149	65	30	8.6
DEHP-d4	283	153	20	8.6
DEHP-d4	153	125	17	8.6
DEHP-d4	153	97	23	8.6
DEHP-d4*	153	69	29	8.6

Quantification of transformation products of DEHP

MEHP, phthalic acid, and phthalic anhydride were quantified using a LC 1290 Infinity II coupled to a triple quadrupole MS 6470 LC/TQ (both Agilent Technologies, Santa Clara, U.S.).

A HSS T3 column (1.8 µm VanGuard FIT, 2.1 x 100mm, Acquity Premier, Waters, Milford, U.S.) and a guard column (2.1 mm x 5 mm, Acquity, Waters, Milford, U.S.) were used for chromatographic separation. The injection volume was 1 µL. Ultra-pure water (phase A) and methanol with 0.1 % (v/v) formic acid (phase B) served as mobile phases. The column temperature was 40 °C and the flow was 0.3 mL min^{-1}. The elution started with 5 % phase B which then increased to 95 % (0–20 min). Phase B further increased to 100 % (20–25 min), held for 1 min (25–26 min), and then decreased to 5 % and equilibrated for 2 min. The source parameters were as follows: the gas temperature was 240 °C, the gas flow was 5 L min^{-1}, the nebulizer pressure was 30 psi and the sheath gas temperature was 230 °C. All transitions were measured in positive ion mode (**Table S1.2**).

Table S1.2. MS parameters for the quantification of the transformation products. The transitions used as quantifiers are indicated by an asterisk.

Compound	Precursor ion (m/z)	Product ion (m/z)	Fragmentor (V)	Collision energy (V)	Retention time (min)
MEHP*	279.2	149	82	18	18.4
MEHP	149	65	82	18	18.4
Phthalic acid	167	78	122	15	6.0
Phthalic acid	149	121	122	15	6.0
Phthalic acid	149	93.1	122	19	6.0
Phthalic acid*	149	65.2	122	27	6.0
Phthalic acid-d4	171	127	122	15	6.0
Phthalic acid-d4	153	125	122	15	6.0
Phthalic acid-d4	153	97	122	19	6.0
Phthalic acid-d4*	153	69	122	27	6.0
Phthalic anhydride	149	121	122	15	8.4
Phthalic anhydride	149	93.1	122	19	8.4
Phthalic anhydride*	149	65.2	122	27	8.4

S2 Characterization of the PVC microplastics before and after photoaging

S2.1 Mass of the PVC microplastics before and after photoaging

The mass of the PVC microplastics was determined before and after 24 and 48 d of photoaging by weighing 20 PVC microplastic pellets and calculating the average mass. The release of small (volatile) molecules (e.g., water, CO, CO_2 or, HCl) led to weight loss of the PVC microplastics with increasing exposure to UV light (**Figure S2.1**).[1] HCl has been reported to account for >95 % of released volatile molecules during PVC degradation.[2] The average weight of a single PVC microplastic pellet decreased from 27.7 ± 0.222 mg for $PVC_{Pristine}$ to 27.0 ± 0.278 mg and 26.7 ± 0.268 mg for PVC_{24d} and PVC_{48d}, respectively. Volatile molecules released from the PVC microplastics during photoaging were not quantified.

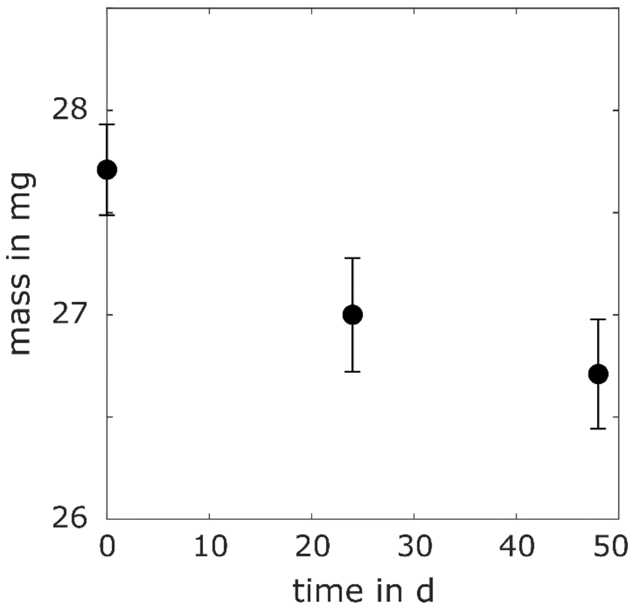

Figure S2.1. Weight loss of PVC microplastics. The average mass of PVC microplastic pellets (mg, y-axis) versus the time of exposure to UV light (days, x-axis) is shown. Error bars represent one standard deviation (n= 20) calculated using Gaussian error propagation.

To ascertain that the mean mass of $PVC_{Pristine}$, PVC_{24d}, and PVC_{48d} differed significantly, a one-way ANOVA analysis was conducted. The significance level alpha was set to 0.05. The results

demonstrate that the mass of the three PVC microplastics differed significantly (p= 8.74* 10^{-17}, **Table S2.1**).

Table S2.1. Significance analysis of the mass of the PVC microplastics before and after photoaging. The mean mass of $PVC_{Pristine}$, PVC_{24d}, and PVC_{48d} as well as the standard deviation (n= 20) and variance are provided. Additionally, the results from the one-way ANOVA analysis including the F-value, the critical F-value (F_{crit}) and the p-value are given.

				ANOVA		
	Mean mass (mg)	Std	Variance	F-value	F_{crit}	p-value
$PVC_{Pristine}$	27.7	0.222	0.0520	75.8	3.16	8.74* 10^{-17}
PVC_{24d}	27.0	0.278	0.0814			
PVC_{48d}	26.7	0.268	0.0758			

S2.2 Appearance of the PVC microplastics before and after photoaging

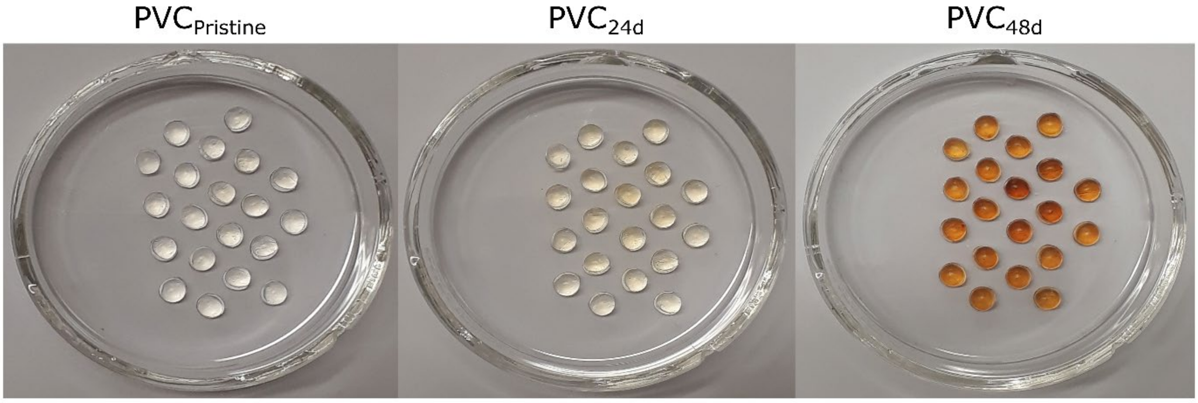

Figure S2.2.1. PVC microplastics before ($PVC_{Pristine}$) and after 24 and 48 d of exposure to UV light (PVC_{24d} and PVC_{48d}, respectively).

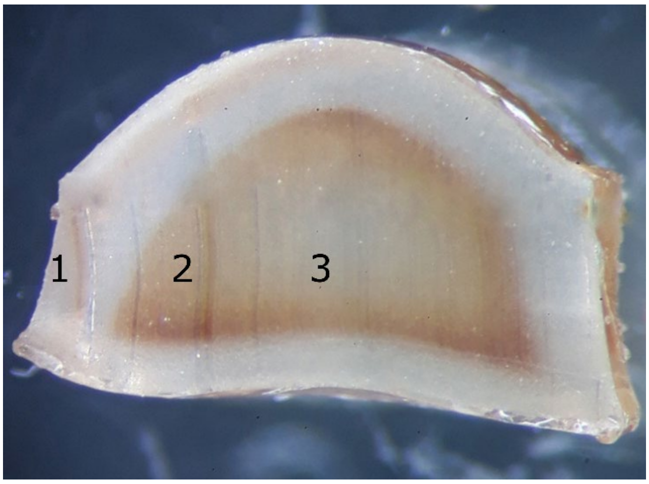

Figure S2.2.2. Cross section of a PVC_{48d} microplastic pellet. The numbers indicate the spots of the TOF-SIMS measurements, which were also applied to the measurements of $PVC_{Pristine}$ and PVC_{24d} (**Figure S2.5**): 1= edge, 2= middle, 3= center. Due to the applied force during cutting, the pellet partly lost its spherical shape and became flat.

S2.3 Surface functional groups of the PVC microplastics

To investigate the surface functional groups of the pristine and aged PVC microplastics, Attenuated Total Reflectance-Fourier Transform Infrared Spectroscopy (ATR-FTIR) was used. Fourier-transform infrared (FTIR) spectra of the PVC microplastics were obtained from 370 cm^{-1} to 4000 cm^{-1} using a Tensor 27 FTIR spectrometer (Bruker, Vienna, Austria). The spectrometer was equipped with a middle-infrared (MIR) light source, a KBr beam splitter, and a DLaTGS detector. The sample and background spectra were obtained using 32 scans at a spectral resolution of 4 cm^{-1}. The PVC microplastic pellets were pressed on the 2 x 3 mm diamond window of a MVP 2 diamond attenuated total reflectance (ATR) accessory (Harrick Scientific, Pleasantville, NY, US). Background spectra were obtained from the empty ATR unit. Spectra were obtained for three sample replicates. Data were evaluated using OPUS 5.5 software (Bruker, Vienna, Austria). For the measurement of the cross sections, the PVC microplastics were cut into halves and the inside of the pellet was pressed on the diamond window.

ATR-FTIR spectra of the top and bottom side confirmed uniform aging of the PVC microplastics in the UV-chamber (**Figure S2.3.1**). Photoaging led to changes of the surface functional groups of the PVC microplastics (**Figure S2.3.2**). An increase in C=O and -OH groups, corresponding to the absorbance at 1610–1830 cm^{-1} and 3000–3500 cm^{-1}, respectively, from $PVC_{Pristine}$ to PVC_{24d} and PVC_{48d} pointed out the formation of oxygen-containing functional groups in the polymer.[3] Changes of the polymer structure with increasing exposure to UV light, e.g., chain scission, were revealed by decreasing absorbance at 2860 cm^{-1} and at 700 cm^{-1} corresponding to $-CH_2$ groups of the polymer chain; a decrease of $-CH_3$ groups was derived from decreasing absorbance at 1377 and 1447 cm^{-1}.[4] Decreasing absorbance at 617 cm^{-1} (corresponding to C-Cl groups) demonstrated the dechlorination of PVC with increasing UV irradiation (**Figure S4**).[5] ATR-FTIR spectra of the cross sections of pristine and photoaged PVC microplastics were similar since the formation of oxidation species was limited to the surface layer and polyene species were not visible in the IR spectra (**Figure S2.3.1**).[6] Besides effects of photoaging on the polymer, ATR-FTIR spectra of pristine and photoaged microplastics indicated the transformation of DEHP at the particle surface (**Figure S2.3.2**). With increasing exposure time of PVC microplastics to UV light, the intensity of the signal at 1072 and 1125 cm^{-1} (representing aromatic $O-CH_2$ groups of DEHP) and at 1280 cm^{-1} (representing conjugated aromatic ester COO groups of DEHP) became less distinct, indicating the transformation of DEHP to smaller molecules, e.g., monoesters.[4,7] The formation of phthalic acid was indicated by the increasing intensity and broadening signal of the C=O groups (1610–1830 cm^{-1}).[7,8]

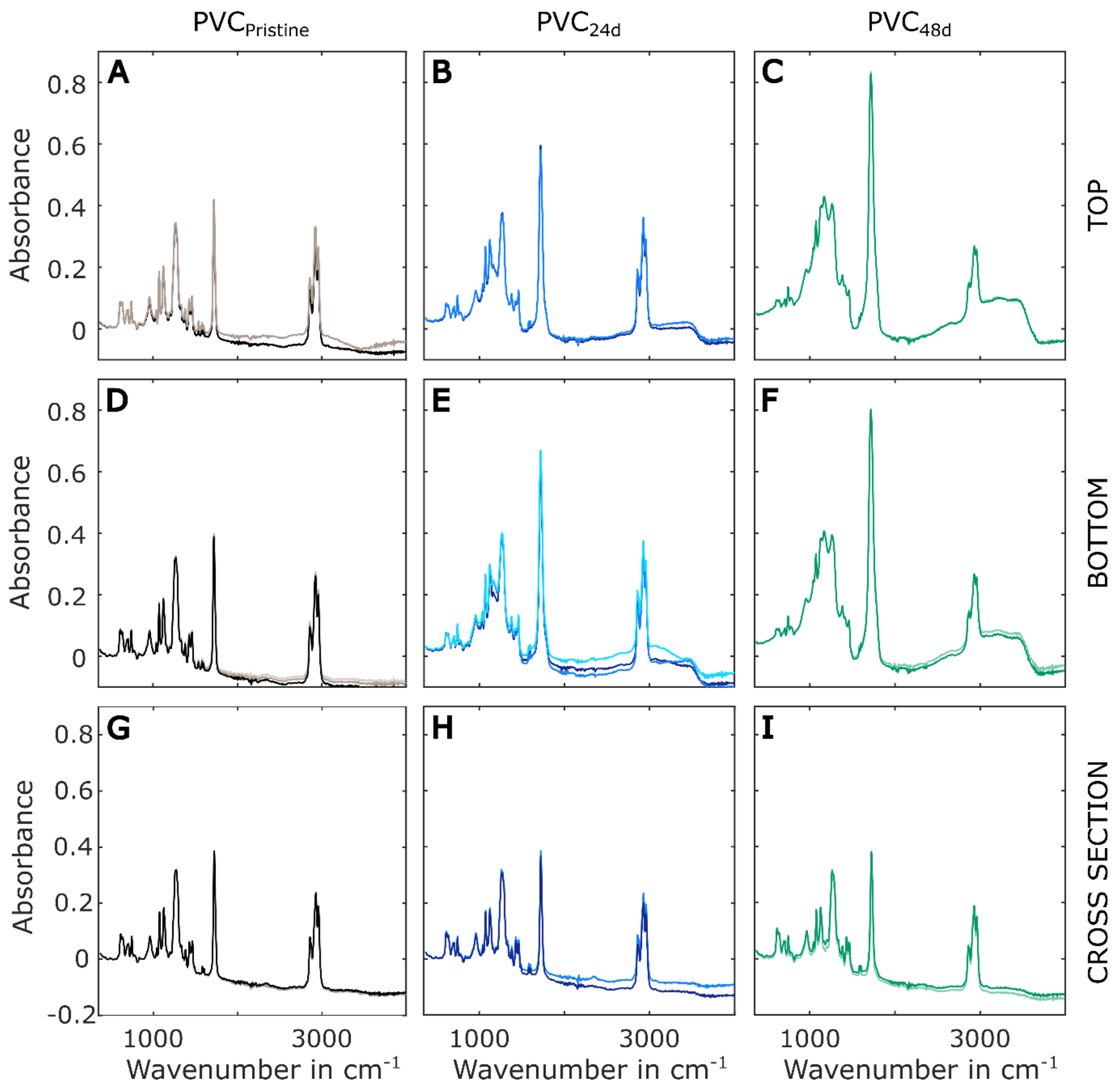

Figure S2.3.1. ATR-FTIR spectra of the PVC microplastics. The absorbance (y-axis) at each wavenumber (cm^{-1}, x-axis) of the top side (A–C), bottom side (D–F), and cross section (G–I) of $PVC_{Pristine}$, PVC_{24d} and PVC_{48d} is shown. The measurements were performed with two to three sample replicates, which are shown in different color shades.

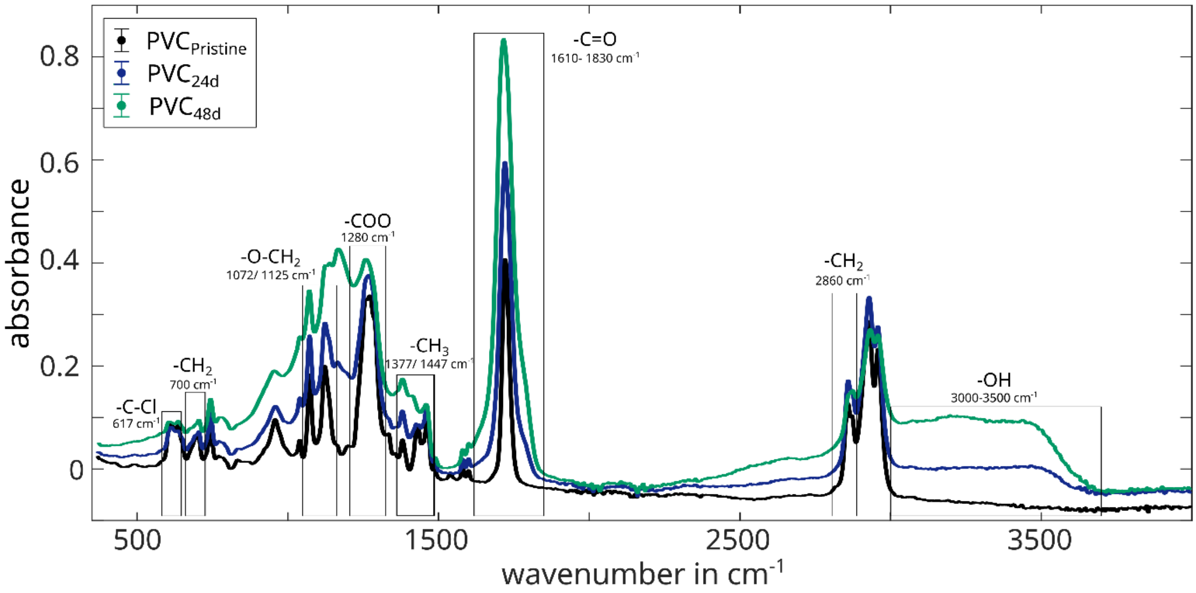

Figure S2.3.2. ATR-FTIR spectra of PVC microplastics. The absorbance (y-axis) at each wavenumber (cm^{-1}, x-axis) for $PVC_{Pristine}$, PVC_{24d} and PVC_{48d} is shown. Selected functional groups are highlighted.

The increase in C=O groups was as used as a proxy for the degree of surface oxidation. To analyze the relationship between surface oxidation and the time-dependent leaching of DEHP from $PVC_{Pristine}$, PVC_{24d} and PVC_{48d}, the carbonyl index I_{CO} (**Equation 1**) was plotted against the leaching rate, and against the modeled log $K_{PVC/W}$ (**Figure S2.3.3**). A linear regression line (y= 0.00177x + 0.123) could well-describe the relationship between the leaching rate and I_{CO} (R^2= 0.893, standard error= 0.0172). The relationship between log $K_{PVC/W}$ and I_{CO} could also be adequately described using a linear regression line (y= -0.0147x + 8.69, R^2= 0.867, and standard error = 0.161).

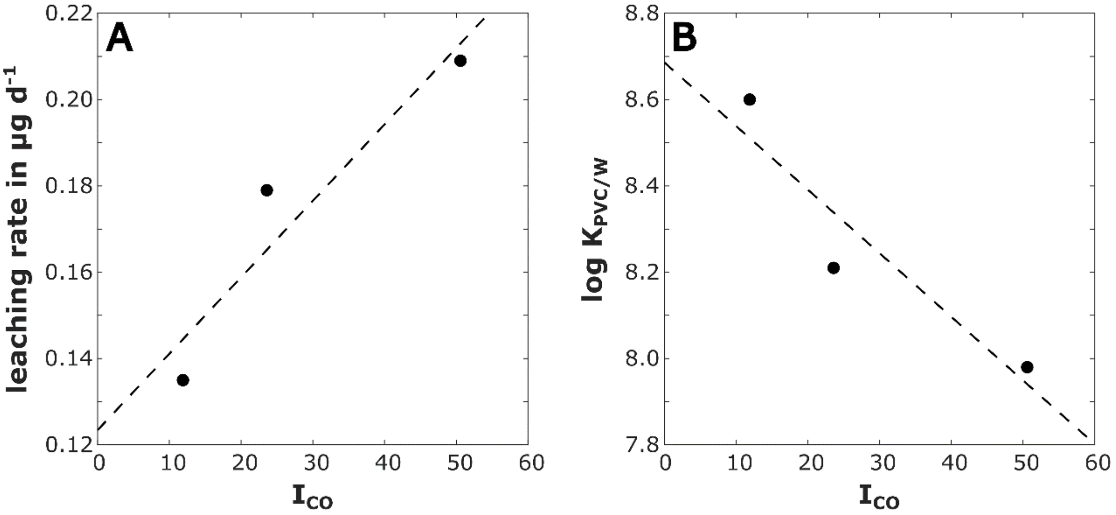

Figure S2.3.3. Relationship between the surface oxidation of the PVC microplastics and the time-dependent leaching of DEHP. A) The leaching rates of $PVC_{Pristine}$, PVC_{24d} and PVC_{48d} (µg d^{-1}) plotted versus the carbonyl index (I_{CO}) calculated from the ATR-FTIR spectra (**Equation 1**) are shown (black circles). B) The logarithmic partition coefficient (log $K_{PVC/W}$) for the leaching of DEHP from $PVC_{Pristine}$, PVC_{24d} and PVC_{48d} versus I_{CO} is shown (black circles). Linear regression lines were used to describe the data (dashed black lines).

S2.4 Initial content of DEHP and transformation products of the PVC microplastics

The DEHP content of the PVC microplastics was determined following the standard operation procedure for consumer product safety.[9] 50 mg of $PVC_{Pristine}$, PVC_{24d} and PVC_{48d} were dissolved in 5 mL of THF using 40 mL glass centrifuge vials. The vials were placed on a horizontal shaker at 125 rpm for 30 min until complete dissolution of the sample. 10 mL of n-hexane were added, and the vials were again placed on the horizontal shaker. Afterwards, the vials were centrifuged at 1000 G at 20 °C for 30 min to allow the polymer to precipitate. Since a high DEHP content was expected, samples were diluted by spiking 25 µL of the clear supernatant into 2.5 mL of n-hexane. 980 µL of the diluted sample were spiked with 20 µL of deuterated DEHP-d_4 standard (corresponding to 1 µg DEHP-d_4) for the quantification of DEHP. The samples were measured using GC-MS/MS (see **S1**). To measure the content of

transformation products of the photoaged microplastics, 80 µL and 35 µL of the clear supernatant were spiked into 2.5 mL isopropanol. 980 µL of the diluted sample were spiked with 20 µL of deuterated phthalic acid-d4 standard (corresponding to 1 µg phthalic acid-d_4) for the quantification of the MEHP, phthalic acid, and phthalic anhydride. The samples were measured using LC-MS/MS (see **S1**). The content of DEHP and of the transformation products of the photoaged PVC microplastics was determined using triplicates. Results are given in **Table 1**.

To ascertain that the mean DEHP content in $PVC_{Pristine}$, PVC_{24d}, and PVC_{48d} (**Table 1**) differed significantly, and to ascertain that the mean content of each transformation product in PVC_{24d} and PVC_{48d} differed significantly, one-way ANOVA analyses were conducted. The significance level alpha was set to 0.05. The results are provided in **Table S2.4**.

Table S2.4. Significance analysis of the content of DEHP and its transformation products in the PVC microplastics. The mean content of DEHP in $PVC_{Pristine}$, PVC_{24d}, and PVC_{48d}, and the mean content of MEHP, phthalic acid, and phthalic anhydride in PVC_{24d}, and PVC_{48d} as well as the standard deviation (n= 3) and variance are given. Additionally, the results from the one-way ANOVA analysis including the F-value, the critical F-value (F_{crit}) and the *p*-value are given.

				ANOVA		
	Mean content (µg)	Std	Variance	F-value	F_{crit}	*p*-value
**DEHP**						
$PVC_{Pristine}$	$32.1 * 10^3$	937	1754435	371	5.79	$3.66 * 10^{-6}$
PVC_{24d}	$19.2 * 10^3$	359	193151			
PVC_{48d}	$13.3 * 10^3$	495	367274			
**MEHP**						
PVC_{24d}	506	23.3	816	448	7.71	$2.95 * 10^{-5}$
PVC_{48d}	$1.3 * 10^3$	50.8	3867			
**Phthalic acid**						
PVC_{24d}	11.0	1.91	5.49	503	7.71	$2.34 * 10^{-5}$
PVC_{48d}	142	8.01	96.2			
**Phthalic anh.**						
PVC_{24d}	0.287	0.0142	$3.04 * 10^{-4}$	900	7.71	$7.35 * 10^{-6}$
PVC_{48d}	1.34	0.0477	$3.41 * 10^{-3}$			

S2.5 Distribution of DEHP in the PVC microplastics

Thermal aging of plasticized PVC can enhance the diffusion and enrichment of plasticizers at the surface of the microplastics and thereby, lead to higher leaching rates.[10] Therefore, the spatial distribution of DEHP in the PVC microplastics was assessed using Time-of-Flight Secondary Ion Mass Spectrometry (TOF-SIMS) measurements. PVC microplastics were cut in halves and placed in clear frozen section compound (VWR international, Montreal, Canada). Samples were gently frozen using liquid nitrogen. Frozen samples were transferred to a 3050 S cryomicrotrome (Leica, Vaughan, Canada) and cut at -20 °C in 60 µm thick slices which were

then thaw-mounted on a glass slide. For each PVC microplastics, sample duplicates were prepared. Samples were analyzed with a PHI nano TOF-SIMS (Physical Electronics, Chanhassen, U.S.). A primary ion beam of 30 keV Bi^{3++} was used. Mass spectra were acquired over a mass range of 0-500 amu in positive-ion mode. To ensure the accuracy of the ion piece identification during each analysis, mass calibration with common organic fragments ($C_2H_5^+$, m/z= 29.04 amu, $C_3H_7^+$, m/z = 43.05 amu, and $C_4H_8^+$, m/z = 56.06 amu) was performed. The spectra for total ions and for DEHP (m/z= 391 amu) were acquired. Although direct concentration measurement using TOF-SIMS is difficult, semi-quantitative analysis using normalized intensities (ratio of the ion count for the compound of interest to the total ion count) is a common strategy to compare the relative concentration of a compound in different samples, given that the same measurement conditions were used.[11,12] Three locations (**Figure S2.2.2**) were analyzed for each PVC microplastics and the relative intensity (calculated as the ratio of the ion count of DEHP to the total ion count times 100) was used for comparison of the samples. The spatial distribution of transformation products of DEHP could not be assessed since the transformation products formed during UV irradiation and the fragments formed during the measurement of DEHP have the same m/z. To ascertain that there was a statistically significant difference between the mean DEHP concentration in the three PVC microplastics, a one-way ANOVA analysis was conducted. The significance level alpha was set to 0.05. The results are provided in **Table S2.5**.

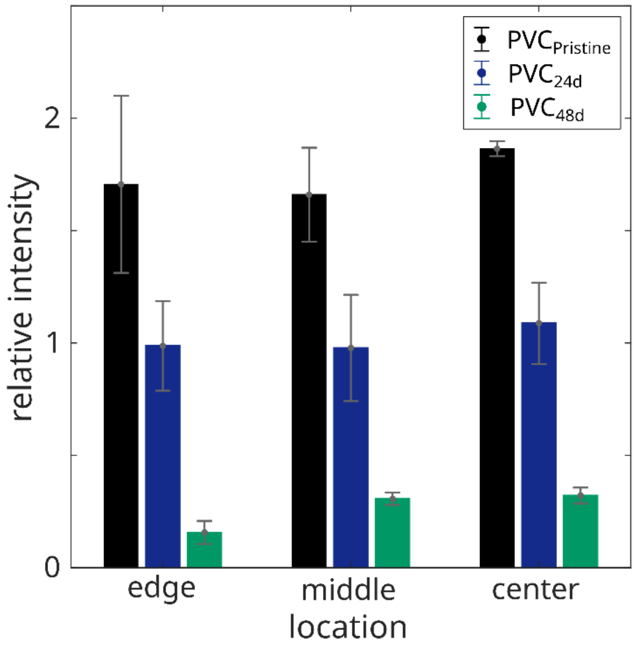

Figure S2.5. Spatial distribution of DEHP in the cross sections of PVC microplastics determined using TOF-SIMS measurements. The relative intensities (y-axis), calculated as the ratio of the ion count of DEHP to the total ion count times 100 at three different locations (x-axis) for $PVC_{Pristine}$, PVC_{24d} and PVC_{48d} are shown. Error bars represent one standard deviation (n= 2).

Table S2.5. Significance analysis of the DEHP concentration determined using Time-of-Flight Secondary Ion Mass Spectrometry (TOF-SIMS) measurements. The mean relative intensity of DEHP in $PVC_{Pristine}$, PVC_{24d}, and PVC_{48d} as well as the standard deviation (n= 6) and variance are provided. Additionally, the results from the one-way ANOVA analysis including the F-value, the critical F-value (F_{crit}) and the *p*-value are given.

				ANOVA		
	Mean intensity	Std	Variance	F-value	F_{crit}	*p*-value
$PVC_{Pristine}$	1.74	0.272	0.089	65.1	3.68	$4.05 * 10^{-8}$
PVC_{24d}	1.02	0.213	0.054			
PVC_{48d}	0.262	0.084	0.008			

S2.6 Surface area of the PVC microplastics

The surface area of the PVC microplastics was determined from N_2 sorption isotherm at 77.3 K using Brunauer–Emmett–Teller (BET) analysis on a Nova 2000e Surface Area and Pore Size Analyzer (Quantachrome Instruments, Boynton Beach, Florida, U.S.). Before the measurement, samples were degassed under vacuum at 20 °C overnight.

S2.7 Glass transition temperature and molar mass distribution

The glass transition temperature of the PVC microplastics was determined using Differential Scanning Calorimetry (DSC) with a Q2000 V24.10 Build 122 (TA Instruments, New Castle, U.S.). For the analysis, 8.5 mg of PVC microplastics were heated to 150 °C at 20 °C min^{-1}.

The molar mass distribution of the PVC microplastics was studied using Gel Permeation Chromatography (GPC). Therefore, 2 mg mL^{-1} of the respective PVC microplastics were dissolved in the eluent. The resulting solutions were filtered through 0.2 µm syringe filters (Sartorius, Göttingen, Germany) prior to the sample injection. The injection volume was 100 µL. Samples were measured using a modular GPC system with two PLgel 10 µm MIXED-B, 7.5 mm x 300 mm columns (Agilent Technologies, Santa Clara, U.S.) and a refractive index detector (Agilent 1100, Agilent Technologies, Santa Clara, U.S.). The column temperature was set to 35 °C and with a flow rate of 1 mL min^{-1}. A mixture of THF with 0.1 % trifluoroacetic acid served as the mobile phase. From the GPC spectra the weight-averaged molecular weight (M_w) and the number-averaged molecular weight (M_n) were determined.

S3 Stability of transformation products in water

To ensure the transformation products measured in the framework of the leaching experiments resulted from photoaging and not from further transformation of DEHP and transformation products in water, a recovery experiment was performed over 24 days. Therefore, 1 µg of MEHP, phthalic acid, and phthalic anhydride was spiked in a 1.5 mL brown glass vial and placed on a horizontal shaker at the same conditions used for leaching

experiments: at a shaking speed of 125 rpm, 20 °C and in dark. The stability of each transformation product was investigated separately. After defined time intervals (0-1-3-5-9-15-19-24 d), MEHP, phthalic acid, and phthalic anhydride were quantified using LC-MS/MS (**S1**). DEHP has a hydrolysis half-life of 2000 years.[13] Since transformation products were not measured in the leaching experiments using $PVC_{Pristine}$, the transformation of DEHP to MEHP, phthalic acid and phthalic anhydride could be ruled out.

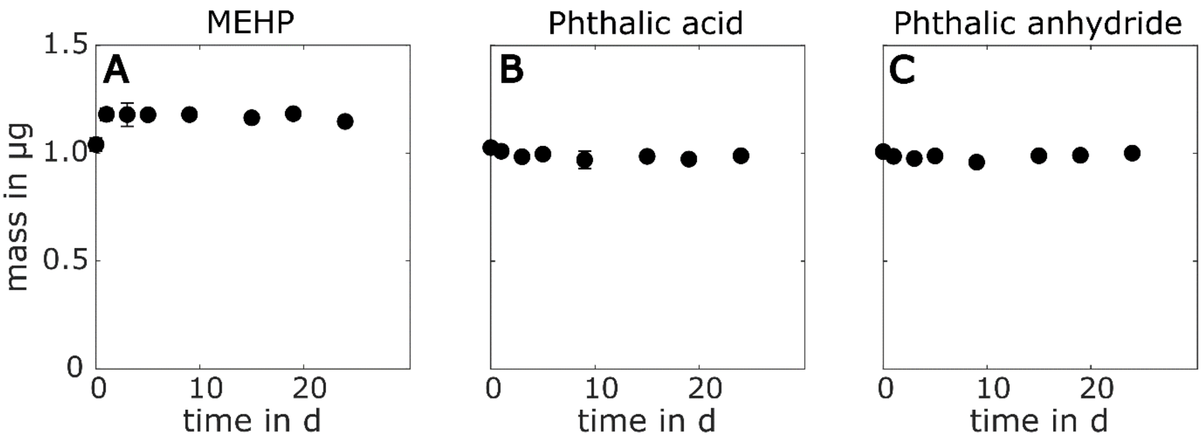

Figure S3. Stability of the transformation products of DEHP in water. The mass (µg, y-axis) of A) MEHP, B) phthalic acid, and C) phthalic anhydride at the respective sampling time (days, x-axis) is shown. Error bars represent one standard deviation (n= 3) calculated using Gaussian error propagation.

To determine whether the mean concentrations of MEHP, phthalic acid, and phthalic anhydride differed significantly over time, one-way ANOVA analyses were conducted. The significance level alpha was set to 0.05. The results are provided in **Table S3**. The concentration of phthalic acid remained constant (p= 0.187) for the entire duration of the experiment. The concentration of MEHP was constant between 1 and 24 d (p= 0.901). The concentration at 0 d was slightly lower, probably due to an error in spiking the standard (p= 0.0223 for the concentration of MEHP between 0 and 24 d). Similarly, the concentration of phthalic anhydride was constant between 0 and 5 d, and between 15 and 24 d (p= 0.0594). The concentration at 9

d was slightly lower, probably also due to an error in spiking the standard (*p*= 0.00177 for the concentration of phthalic anhydride between 0 and 24 d).

Table S3. Significance analysis of the concentration of transformation products over time. The mean concentration of MEHP, phthalic acid and phthalic anhydride, as well as the standard deviation (n= 3) and variance are provided. The results from the one-way ANOVA analysis including the F-value, the critical F-value (F_{crit}) and the *p*-value are given.

				ANOVA		
	Mean conc. (ng mL^{-1})	Std	Variance	F-value	F_{crit}	*p*-value
**MEHP**						
0 d*	1061	10.1	203			
1 d	1180	28.9	1256			
3 d	1179	54.4	4445			
5 d	1178	12.0	215	0.344	2.92	0.901
9 d	1179	18.4	509			
15 d	1164	12.4	229			
19 d	1183	18.0	484			
24 d	1147	7.65	117			
**Phthalic acid**						
0 d	1027	13.6	277			
1 d	1010	9.05	123			
3 d	984	17.6	462			
5 d	996	17.2	446	1.67	2.66	0.187
9 d	970	40.3	2437			
15 d	986	15.9	380			
19 d	973	20.6	635			
24 d	989	15.3	351			
**Phthalic anh.**						
0 d	1008	9.92	148			
1 d	986	3.52	18.6			
3 d	976	6.90	71.5			
5 d	988	4.27	27.3	2.69	2.85	0.0594
9 d*	959	7.50	84.3			
15 d	989	8.31	104			
19 d	991	11.7	205			
24 d	1001	13.1	256			

* not included in the ANOVA analysis

S4 Regression analysis of time-dependent leaching curves

The time-dependent leaching curves for DEHP from $PVC_{Pristine}$, PVC_{24d}, and PVC_{48d} were fitted using linear regression lines (**Figure 1**). To ascertain that these regression lines could adequately describe the experimental data and that the found coefficients were significant, a regression analysis was conducted. The significance level alpha was set to 0.05. The results are provided in **Table S4.1**. The high R^2 values (≥ 0.947) and low *p*-values (≤ $4.82*10^{-5}$) indicated that the regression model could well describe the data. The low *p*-values for the coefficient for x further indicated that the slopes of the regression lines were significant.

Table S4.1 Regression analysis of the time-dependent leaching curves of DEHP from $PVC_{Pristine}$, PVC_{24d}, and PVC_{48d} (**Figure 1**). For the regression analysis R^2 and the standard error are given. The results from the ANOVA analysis are provided for the regression model including the F-value and the *p*-value and for the coefficient for x (slope) including the coefficient, the *p*-value, the lower 95% confidence interval (LC), and the upper 95% confidence interval (UC)).

	Regression statistics		ANOVA					
			Regression		x			
	R^2	Std error	F-value	*p*-value	coeff.	*p*-value	LC	UC
**DEHP (µg)**								
$PVC_{Pristine}$ y=0.135x+0.199	0.998	0.204	2412	$4.78*10^{-9}$	0.135	$4.78*10^{-9}$	0.129	0.142
PVC_{24d} y=0.179x+ 2.64	0.947	1.28	106	$4.82*10^{-5}$	0.179	$4.82*10^{-5}$	0.137	0.222
PVC_{48d} y=0.209x+ 6.83	0.953	1.40	121	$3.32*10^{-5}$	0.209	$3.32*10^{-5}$	0.163	0.256

In contrast to DEHP, the time-dependent leaching curves of MEHP, phthalic acid, and phthalic anhydride from PVC_{24d} and PVC_{48d} were not linear over the entire duration of the experiment (**Figure 2**). Leaching was faster at the beginning of the experiment (1–16 d) and slowed down towards later time points (**Table 2**). To estimate the leaching rates at early and late time points,

the data were fitted using two linear regression models, one for the time points between 1 and 16 d and one for the later time points (**Table S4.2**). The high R^2 values (in most cases ≥ 0.900) and the low *p*-values (in most cases <0.05) indicated that the regression model could well describe the data. Deviations of the regression model from the experimental data can be attributed to changes in the leaching rates from one time point to another due to the heterogeneous polymer structure resulting from photoaging. The mass transfer processes of the transformation products in the PVC microplastics are discussed in more detail in the main manuscript.

Table S4.2 Regression analysis of the time-dependent leaching curves of MEHP, phthalic acid and phthalic anhydride from PVC_{24d}, and PVC_{48d} (**Figure 2** and **Table 2**). For the regression analysis R^2 and the standard error are given. The results from the ANOVA analysis are provided for the regression model including the F-value and the *p*-value and for the coefficient for x (slope) including the coefficient, the *p*-value, the lower 95% confidence interval (LC), and the upper 95% confidence interval (UC). Data for MEHP are given in µg, while data for phthalic acid and phthalic anhydride are in ng.

	Regression statistics		ANOVA					
			Regression		x			
	R^2	Std error	F-value	*p*-value	coeff.	*p*-value	LC	UC
**MEHP (µg)**								
PVC_{24d} 1-16 d	0.771	7.57	10.1	0.0501	2.03	0.0501	-0.0024	4.06
PVC_{24d} 16-80 d	0.993	0.363	276	0.0036	0.125	0.0036	0.0929	0.158
PVC_{48d} 1-16 d	0.869	4.57	20.0	0.0209	1.72	0.0209	0.494	2.94
PVC_{48d} 16-80 d	0.934	1.99	28.3	0.0336	0.221	0.0336	0.0421	0.399
**Phth. acid (ng)**								
PVC_{24d} 1-16 d	0.676	252	6.27	0.0874	53.1	0.0874	-14.4	121
PVC_{24d} 16-80 d	0.123	203	0.282	0.649	2.24	0.649	-15.9	20.4
PVC_{48d} 1-16 d	0.934	405	42.5	0.00733	223	0.00733	114	331
PVC_{48d} 16-80 d	0.949	587	37.3	0.0258	74.6	0.0258	22.0	127
**Phth. anh. (ng)**								
PVC_{24d} 1-16 d	0.980	195	147	0.00121	199	0.00121	147	251
PVC_{24d} 16-80 d	0.961	200	49.4	0.0197	29.2	0.0197	11.3	47.0
PVC_{48d} 1-16 d	0.967	166	86.6	0.00262	130	0.00262	85.7	174.7
PVC_{48d} 16-80 d	0.977	285	86.0	0.0114	54.9	0.00114	29.4	80.4

S5 Degradation of PVC induced by UV light

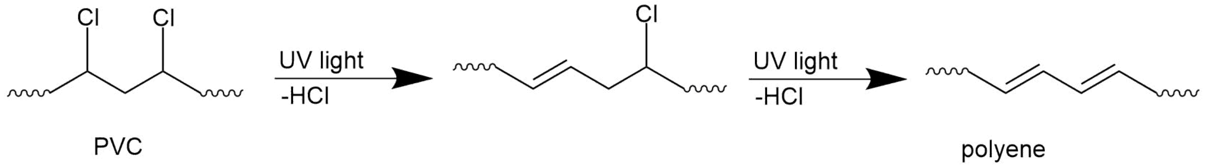

Figure S5. Dechlorination of PVC under UV light and the formation of polyene.[14]

S6 Transformation pathway of DEHP induced by UV light

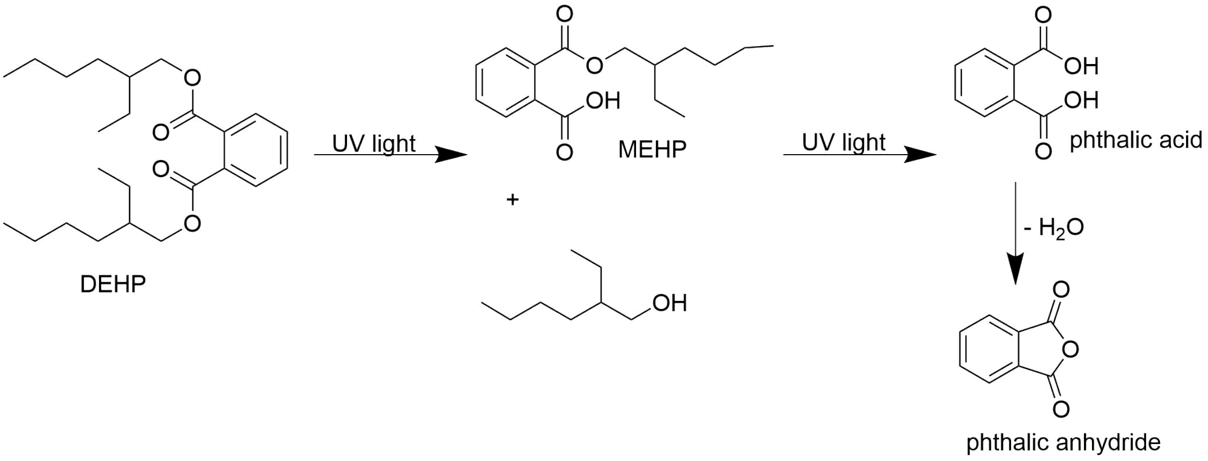

Figure S6. Transformation of DEHP to MEHP, phthalic acid, and phthalic anhydride under UV light.[7]

SUPPLEMENTARY REFERENCES

(1) Feldman, D. Polymer Weathering: Photo-Oxidation. *J. Polym. Environ.* **2002**, *10*, 163–173. https://doi.org/10.1023/A:1021148205366.

(2) Stromberg, R. R.; Straus, S.; Achhammer, B. G. Thermal Decomposition of Poly(Vinyl Chloride). *J. Polym. Sci.* **1959**, *35*, 355–368. https://doi.org/10.1002/pol.1959.1203512904.

(3) Ouyang, Z.; Zhang, Z.; Jing, Y.; Bai, L.; Zhao, M.; Hao, X.; Li, X.; Guo, X. The Photo-Aging of Polyvinyl Chloride Microplastics under Different UV Irradiations. *Gondwana Res.* **2022**, *108*, 72–80. https://doi.org/10.1016/j.gr.2021.07.010.

(4) Ishiaku, U. S.; Mohd Ishak, Z. A.; Ismail, H.; Nasir, M. The Effect of Di-2-Ethylhexyl Phthalate on the Thermo-Oxidative Ageing of Poly(Vinyl Chloride)/Epoxidized Natural Rubber Blends. *Polym. Int.* **1996**, *41*, 327–336. https://doi.org/10.1002/(SICI)1097-0126(199611)41:3<327::AID-PI618>3.0.CO;2-3.

(5) Wang, C.; Xian, Z.; Jin, X.; Liang, S.; Chen, Z.; Pan, B.; Wu, B.; Ok, Y. S.; Gu, C. Photo-Aging of Polyvinyl Chloride Microplastic in the Presence of Natural Organic Acids. *Water Res.* **2020**, *183*, 116082. https://doi.org/10.1016/j.watres.2020.116082.

(6) Gardette, J. L.; Gaumet, S.; Philippart, J. L. Influence of the Experimental Conditions on the Photooxidation of Poly(Vinyl Chloride). *J. Appl. Polym. Sci.* **1993**, *48*, 1885–1895. https://doi.org/10.1002/app.1993.070481102.

(7) Hankett, J. M.; Collin, W. R.; Chen, Z. Molecular Structural Changes of Plasticized PVC after UV Light Exposure. *J. Phys. Chem. B* **2013**, *117*, 16336–16344. https://doi.org/10.1021/jp409254y.

(8) Mishra, M. K. Fourier Transform Infrared Spectrophotometry Studies of Chromium Trioxide-Phthalic Acid Complexes. *Chem. Sci. Trans.* **2016**, *5*, 770–774. https://doi.org/10.7598/cst2016.1260.

(9) U.S. Consumer Product Safety Commision (CPSC). Standard Operation Procedure for Determination of Phthalates. **2010**, 1–8.

(10) Calò, E.; Greco, A.; Maffezzoli, A. Effects of Diffusion of a Naturally-Derived Plasticizer from Soft PVC. *Polym. Degrad. Stab.* **2011**, *96*, 784–789.

https://doi.org/10.1016/j.polymdegradstab.2011.02.012.

(11) Bai, S.; Yu, P.; Li, C.; Wen, S.; Ding, Z. Depression of Pyrite in a Low-Alkaline Medium with Added Calcium Hypochlorite: Experiment, Visual MINTEQ Models, XPS, and ToF–SIMS Studies. *Miner. Eng.* **2019**, *141*, 105853. https://doi.org/10.1016/j.mineng.2019.105853.

(12) Trzyna-Sowa, M.; Berchenko, N.; Dziawa, P.; Cebulski, J. Molecular Speciation Analysis of Oxidized Metal Surfaces by TOF SIMS. *Appl. Surf. Sci.* **2022**, *577*, 151855. https://doi.org/10.1016/j.apsusc.2021.151855.

(13) Staples, C. A.; Peterson, D. R.; Perkerton, T. F.; Adams, W. J. The Environmental Fate of Phthalate Esters: A Literature Review. *Chemosphere* **1997**, *35*, 667–749. https://doi.org/10.1016/S0045-6535(97)00195-1.

(14) Gewert, B.; Plassmann, M. M.; MacLeod, M. Pathways for Degradation of Plastic Polymers Floating in the Marine Environment. *Environ. Sci. Process. Impacts* **2015**, *17*, 1513–1521. https://doi.org/10.1039/c5em00207a.
